# Optimised stress – intensification of pyocyanin production with zinc oxide nanoparticles

**DOI:** 10.1186/s12934-024-02486-y

**Published:** 2024-07-27

**Authors:** Joanna Honselmann genannt Humme, Kamila Dubrowska, Bartłomiej Grygorcewicz, Marta Gliźniewicz, Oliwia Paszkiewicz, Anna Głowacka, Daniel Musik, Grzegorz Story, Rafał Rakoczy, Adrian Augustyniak

**Affiliations:** 1 Department of Chemical and Process Engineering, Faculty of Chemical Technology and Engineering, West Pomeranian University of Technology in Szczecin, Piastów Avenue 42, Szczecin, 71-065 Poland; 2 Department of Forensic Genetics, Faculty of Medicine and Dentistry, Pomeranian Medical University in Szczecin, Powstańców Wielkopolskich 72, Szczecin, 70-111 Poland; 3 Department of Environmental Engineering, Faculty of Civil and Environmental Engineering, West Pomeranian University of Technology in Szczecin, Piastów Avenue 50a, Szczecin, 70-311 Poland; 4 ESC Global, Sp. z o.o., Słoneczny Sad 4F, 72-002 Dołuje, Poland; 5 Center for Advanced Materials and Manufacturing Process Engineering (CAMMPE), Piastow Avenue 42, Szczecin, 71-065 Poland; 6 Chair of Building Materials and Construction Chemistry, Technische Universität Berlin, Gustav- Meyer-Allee 25, 13355 Berlin, Germany

**Keywords:** Bioprocessing, Design of experiment (DoE), Nanomaterials, Phenazines, Stimulation

## Abstract

**Background:**

Pyocyanin is a blue pigment produced by *Pseudomonas aeruginosa.* Due to its unique redox properties over the last decade, it has gained more and more interest as a utile chemical. Nevertheless, it remains a rather costly reagent. It was previously shown that the production of pyocyanin can be enhanced by employing various methods. Among them are using statistical methods for planning the experiments or exposing bacterial cultures to stressors such as nanoparticles dosed in sublethal concentrations, e.g. zinc oxide nanoparticles.

**Results:**

The Design of Experiment (DoE) methodology allowed for calculating the optimal process temperature and nanoparticle concentration to intensify pyocyanin production. Low concentrations of the nanoparticles (6.06 µg/mL) and a temperature of 32℃ enhanced pyocyanin production, whereas higher concentrations of nanoparticles (275.75 µg/mL) and higher temperature stimulated biomass production and caused the abolishment of pyocyanin production. Elevated pigment production in zinc oxide nanoparticles-supplemented media was sustained in the scaled-up culture. Conducted analyses confirmed that observed stimulation of pyocyanin production is followed by higher membrane potential, altered gene expression, generation of reactive oxygen species, and accumulation of zinc in the cell’s biomass.

**Conclusions:**

Pyocyanin production can be steered using ZnO nanoparticles. Elevated production of pyocyanin due to exposure to nanoparticles is followed by the number of changes in physiology of bacteria and is a result of the cellular stress. We showed that the stress response of bacteria can be optimised using statistical methods and result in producing the desired metabolite more effectively.

**Supplementary Information:**

The online version contains supplementary material available at 10.1186/s12934-024-02486-y.

## Background

*Pseudomonas aeruginosa* is a well-known and widely studied microorganism inhabiting ubiquitous environments. Despite being a notorious pathogen, *P. aeruginosa* produces valuable metabolites, e.g., pigments, rhamnolipids, polymers, etc. [1–6]. Phenazines, including pyocyanin, and siderophores, such as pyoverdine or pyochelin, are included in the first group. The most studied example of pseudomonas-derived phenazine is pyocyanin, which changes colour at different pH values and redox states when excreted into the medium.

In a recently published review paper, we briefly summarised why it is worth modulating pyocyanin production and underlined the potential positive outcomes that can be obtained using pyocyanin [7]. Among them, the most promising uses of pyocyanin seem to be its application in anticancer and antimicrobial therapy, as a biopesticide, or for generating electricity in microbial fuel cells. Even though the chemical synthesis of pyocyanin is known [8], the vendors declare that *P. aeruginosa* produced their product. Moreover, pyocyanin remains a rather costly chemical (prices starting from over 50 euros per 5 mg) [7]. Therefore, several methods of the stimulation of pyocyanin production were presented, including the use of chemical or physical stressors that can lead to higher pigment production. One such stressor is nanoparticles. For example, zinc oxide nanoparticles (ZnO NPs) are well-known for their antibacterial and bacteriostatic properties. For that reason, they are widely used in different branches of technology (e.g., food packaging, sunscreens, or electronics) [9–12]. However, the sublethal doses of zinc oxide (ZnO) nanoparticles (NPs) may have different physiological effects on the tested microorganisms, including stimulating specific properties. Therefore, such a phenomenon could be used to improve biotechnological processes. In our previous work [13], we proved that using nanometric ZnO can affect the physiology and pigment production of *P. aeruginosa* based on the concentration used. It prompted the question of whether the observed phenomenon can be further optimised. The statistical planning of experiments, namely Design of Experiment (DoE), is widely utilised in many branches of science, including optimising biochemical processes. Several research groups applied this method to optimise pyocyanin production [14–19]. The experimental factors covered temperature, pH, agitation, incubation time, inoculum size, and various medium ingredients. For example, Ozdal et al. optimised the concentration and time required to add the oxygen vector to the medium (i.e., hexane) [15].

Here, we aimed to engineer the pyocyanin production process using an in-depth approach to optimise the pyocyanin production in the system stimulated by ZnO nanoparticles and operating under optimised process temperature. In order to achieve that goal, we have created and executed a DoE plan and described the physiological state of cells to indicate the probable stress response mechanism underlying the stimulated metabolite production. The secondary goal of our project was to verify the replicability of stimulated production on a larger scale.

## Materials and methods

The research was carried out with the *Pseudomonas aeruginosa* ATCC 27,853 strain as a pyocyanin producer. The strain was kept at -20 ℃ on Cryobank™ (Pro-Lab Diagnostics, Canada) and revived on Trypticase Soy Agar (TSA) medium (Biomaxima, Poland) at 37 ℃. The cultures were prepared for experiments by inoculating 50 mL TSB medium (Biomaxima, Poland) in 250 mL conical flask with *P. aeruginosa* for an overnight culture (37 ℃, 100 rpm), setting optical density (OD) value to 0.5 and inoculating in King’s A broth (20.00 g/L bacteriological peptone, 10.00 g/L K_2_SO_4_, 3.50 g/L MgCl_2_, 1% v/v glycerol) in ratio 1:100 v/v. [13]. All optimization cultures were conducted in a total volume of 15 mL of inoculated King’s A broth (pure in the control cultures or with the addition of ZnO NPs in the tested samples) on Petri dish (90 mm), without agitation, for 72 h.

Zinc oxide nanoparticles (ZnO NPs) used in this research are commercially available at Merck (Darmstadt, Germany) as nanopowder. The used batch of NPs was characterised elsewhere [20]. The size of NPs was estimated to 50 to 300 nm, the surface area was 14.11 m^2^/g and the shape of the particles was from nanorods to spherical nanostructures.Nanomaterials were suspended in distilled water and sonicated for 30 min. The suspensions were streaked on TSA (Tryptic Soya Agar) medium to exclude microbial contamination. The same procedure was applied for micrometric ZnO (Merck, Darmstadt, Germany).

To assess the stability of the nanoparticles, we performed the measurements of the zeta potential of ZnO NPs incubated in deionized water and King’s A medium at two time points – at time zero and after 72 h. The measurements were conducted employing the Litesizer DLS 700 (Anton Paar) apparatus in polycarbonate cuvettes (Anton Paar’s Omega cuvette). Before each measurement, the samples were sonicated for 10 min. Each sample was measured five times and the results were averaged. ZnO NPs suspended in deionized water had a zeta potential of 17.6 ± 0.5 mV at time zero and 13.2 ± 0.2 mV after 72 h. The positive charge of ZnO NPs suspended in water is in line with the report of Kim et al. [21]. However, the zeta potential of ZnO NPs suspended in King’s A medium was characterized by a negative charge, i.e. -18.2 ± 0.4 and − 16.8 ± 0.1, after 0 and 72 h, respectively. It can probably be attributed to the complex composition of the medium. In the case of both deionized water and King’s A medium the obtained zeta potential values were between ± 10 to ± 30 mV which can be interpreted as incipient instability [22]. Moreover, in both cases, the values were lower after 72 h than at time zero. Overall, the results suggest low stability of the used ZnO NPs.

### Outline of the study

**Fig. 1:**
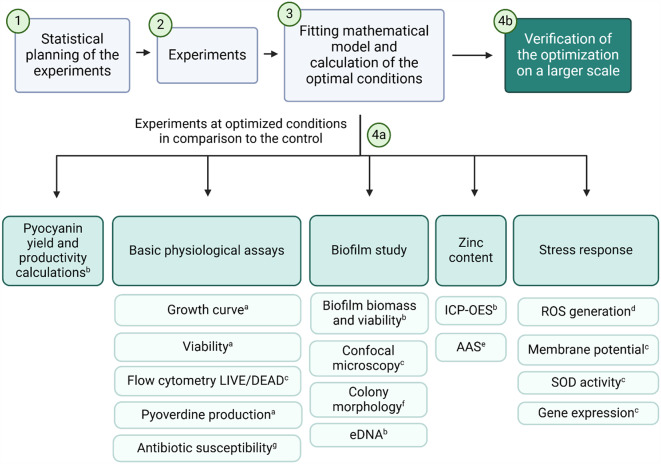
Outline the study with the time points of the experiments conducted. The letters indicate the time points at which the experiment was conducted: (**a**) 0–72 h, (**b**) 24, 48 and 72 h, (**c**) 24 h, (**d**) 12 h, (**e**) 72 h, (**f**) 7 days, (**g**) 6 h

The general outline of the study and experiments that were conducted are summarised in Fig. 1.

### Statistical optimisation of the process

The experiment planning was based on statistical methodology, namely the Design of the Experiment (DoE), using a central composition plan with response surface methodology (RSM) prepared using STATISTICA software (TIBCO Software Inc, version 14.0.0.15). The independently considered variables were the temperature of the process and nanomaterial concentration. The created plan presented in Tab. S1. covered incubation time of 72 h. A wide range of ZnO NPs concentration (9.81–500.00 µg/mL) was proposed as ambiguous effects were previously observed at 37 ℃ [13]. The dependent variables considered in the DoE plan were pyocyanin production (expressed as µg/mL of the culture) and dry biomass (mg/mL of the culture). Additional controls with no ZnO NPs addition were carried out during each experiment. The results were used to fit the mathematical equation using Matlab, which allowed the calculation of the optimal process conditions. The experiments were carried out in three (pyocyanin assay) or two (biomass assay) technical replicates with at least three separate biological repetitions each.

### Scale-up of pyocyanin production

The scale-up was performed by leading the cultures in polypropylene trays with 1 L of King’s A medium (compared to 15 mL in a Petri dish). The pyocyanin was quantified as mentioned below, i.e., chloroform-HCl methodology. The experiment was conducted in three biological and three technical replicates.

### Pyocyanin and biomass quantification

Pyocyanin was extracted employing chloroform-HCl methodology [23, 24]. Shortly, 5 mL of the cell-free culture were mixed with 3 mL of chloroform (60 min on a roller shaker, at room temperature) and centrifuged to separate the phases (6000 rpm, 15 min). Later, 2 mL of chloroform phase were collected in the clean tube and mixed with 1 mL of 0.2 M HCl. The samples were shortly vortexed and centrifuged (6000 rpm, 2 min). The concentration of pyocyanin was measured spectrophotometrically in a microplate reader (520 nm) in the acidic phase and calculated from the standard curve (Supplementary materials, Fig. S1). Biomass was quantified by centrifugation of the cultures (6000 rpm, 15 min), drying the biomass, and weighing on analytical balance.

### Pyocyanin yield and productivity calculations

Glycerol was quantified in culture filtrates (filtered with 0.22 mm PES syringe filter) with a colourimetric Glycerol Assay Kit (Merck, MAK117-1KT). Calculated glycerol concentrations were used for the calculation of the yield (product/substrate) and productivity (product/day) [25], where glycerol is considered a primary carbon source and pyocyanin is a product. Additional samples with micrometric ZnO were tested to compare its effect with ZnO NPs on pyocyanin yield and productivity.

The product/substrate yield coefficient was calculated from the Eq. (1):

1 $$\:{Y}_{PS}=-\frac{dP}{dS}$$

where: *Y*_*PS*_ – product/substrate yield coefficient (µg of pyocyanin/µmol of glycerol), *P* – concentration of the product (pyocyanin; µg/mL), *S* – concentration of the substrate (glycerol; µmol/mL).

The productivity was calculated from the Eq. (2):

2 $$\:{R}_{PS}=\frac{dP}{dt}$$

where: *R*_*PS*_ – productivity (µg/(mL day)), *P* – concentration of the product (pyocyanin; µg/mL), *t* – time (day).

### Basic physiological assays

#### Growth, viability, and pyoverdine monitoring

The optical density (OD, here used to monitor the growth of the culture, *λ* = 600 nm) and viability measurements (aerobic respiration, resazurin reduction assay, *λ*_*ex*_ *=* 520 nm, *λ*_*em*_ *=* 590 nm) were performed at the time points of 2, 4, 6, 8, 10, 12, 24, 48 and 72 h. The maximum growth rate and inflexion point were calculated from the fitted logistic function based on Winsor [26]. Production of pyoverdine (fluorescent siderophore) was monitored fluorometrically directly from the culture at 398/460 nm [27]. Antibiotic susceptibility was tested according to the method described in Supplementary materials (1.3).

#### LIVE/DEAD flow cytometry

The optimised and control cultures were subjected to flow cytometry to assess the viability of the cells. According to the user’s manual, the cells were stained using LIVE/DEAD™ BacLight™ Bacterial Viability Kit for microscopy and quantitative assays (L7012, Thermo Fisher Scientific, USA). SYTO™ 9 is a green fluorescent stain that stains all the cells (both with intact membrane and disrupted one) and propidium iodide is a red-fluorescent stain that stains only the cells with the damaged membrane [28]. The staining was confirmed with epifluorescence microscopy. The measurements were recorded on BD Accuri™ C6 Plus (Franklin Lakes, NJ, USA). Ten thousand events were acquired in each sample. Forward scatter (FSC), side scatter (SSC, threshold = 10^3^), red and green fluorescence were measured. FCS Express (De Novo Software, Pasadena, CA, USA) was used for gating, cytogram generation and statistics.

### Biofilm study

#### Biofilm biomass and viability

The biofilm biomass and its viability were quantified using the standard crystal violet staining method and resazurin reduction test. It was performed in a 96-well polystyrene plate, with all corner wells eliminated from the analysis to avoid the edge effect. Shortly after the incubation, the planktonic cells were removed, the wells were washed twice with PBS buffer (phosphate buffered saline), and the viability of the biofilm was quantified in King’s A medium with 10% v/v of resazurin. Next, the wells were washed twice with PBS, fixed with methanol, stained with 0.1% m/v filtered crystal violet solution, and quantified spectrophotometrically at 595 nm with acetone/ethanol (4:1 v/v). Each type of sample was measured in 15 repetitions in three separate experiments.

#### Extracellular DNA

The content of extracellular DNA (eDNA) in the cultures was measured employing Quant-IT dsDNA Assay (ThermoFisher), as eDNA was previously reported to be intertwined with pyocyanin production [29]. It was measured according to the method described by Das et al. [30] in two biological and three technical replicates. Shortly, the cultures were centrifuged (6000 rpm, 15 min), the supernatants were separated and filtered through 0.22 μm syringe filter to remove any remaining cells. 10 µL of the sample were subjected to the measurement using Quant-IT dsDNA assay by fluorometric measurement and the eDNA concentration was calculated from the standard curve.

#### Confocal laser scanning microscopy

The biofilms of the control and optimised cultures were performed on an 8-well plate and cultivated for 24 h at 32.6 ℃. Later, the biofilm was washed with PBS, stained with LIVE/DEAD™ BacLight™ (ThermoFisher, dyes in ratio 1:1 v/v) for 15 min, and covered with a layer of ProLong™ Glass Antifade Mountant (ThermoFisher). Samples were visualised on Leica Stellaris 5 WLL (Leica, Wetzlar, Germany) and processed on LasX software (Leica, Wetzlar, Germany). Excitation and emission wavelengths were set to match the used fluorophores, i.e. SYTO9 (λ_ex_ = 485 nm, λ_em_ = 498 nm) and Propidium iodide (λ_ex_ = 535 nm, λ_em_ = 617 nm).

#### Zinc content analysis

The zinc concentration in the culture supernatant was quantified by the inductively coupled plasma atomic emission spectroscopy (ICP-OES) method, whereas zinc content in bacterial biomass was analysed using the atomic absorption spectroscopy (AAS) method. Shortly, bacterial cultures were centrifuged to separate the biomass from the supernatant (6000 rpm, 15 min). The supernatant was filtered with a 0.22 μm syringe filter and passed to ICP-OES analysis. The collected biomass was washed twice with PBS buffer to remove any zinc on the outside of the cells and dried at 105 ℃ for 24 h.

An analysis of the zinc concentration in the culture supernatant was carried out with a spectrometer ICP-OES 5100 SVDV Agilent Technologies, USA. Measurements were performed using axial and radial observation. The samples were introduced with an aerosalt nebuliser and a cyclone spray chamber with an ascension pipe. The peristaltic pump performed the transportation of sample solutions. The measurement of the elemental content was carried out following PN-EN ISO 11,885: 2009.

The zinc content in the biomass was determined using Atomic Absorption Spectroscopy (AAS). The weighed samples (1 g each), one containing a zinc additive and the control sample, were completely mineralised in a mineralising mixture (10 mL of perchloric acid and nitric acid in a volume ratio of 1:3, within a temperature range of 110 ℃ to 225 ℃). Subsequently, the solutions were diluted with ultra-pure water to a volume of 50 mL and analysed using an atomic absorption spectrometer iCE 3500 (Thermo Scientific, Bremen, Germany).

### Stress response analyses

#### Membrane potential

Membrane potential is an electrical potential across the membrane that is a source of free energy that can be utilized by the cells to perform mechanical or chemical work. It was reported that membrane potential regulates bacterial physiology, including membrane transport, antibiotic resistance, electrochemical communication and environmental sensing [31]. Considering the fact that pyocyanin is a mediator of electron transfer and is regulated by quorum sensing in *P. aeruginosa*, we decided to check if it is affected by the presence of ZnO NPs.

The membrane potential was assessed using BacLight™ Bacterial Membrane Potential Kit (B34950, Thermo Fisher Scientific, USA) according to the user manual with modifications. The cells were diluted in filtered PBS. Then, CCCP, phenylalanine-arginine β-naphthylamide (PAβN), or both were added to 1 mL of the sample and incubated at room temperature for 5 min. Next, DiOC_2_ and 1mM EDTA were added to the samples and incubated at 37 ℃ for 10 min. Data acquisition and analysis were performed analogically to CLSM assay.

### ROS generation

ROS generation was measured using 2’,7’-dichlorodihydrofluorescein diacetate (DCFH-DA) that penetrates the cells and becomes fluorescent when ROS are present within the cell. The cultures were incubated for 12 h at optimal conditions for pyocyanin production. Then, the culture was centrifuged (6000 rpm, 15 min), washed with PBS, resuspended in King’s A medium containing 50 µM of DCFH-DA, and incubated for 30 min in the dark. After that, the cells were centrifuged (10,000 rpm, 2 min), washed with PBS, and resuspended in PBS. The OD was unified for all the samples, and the fluorescence was measured at ʎ_ex_ = 485 nm and ʎ_em_ = 530 nm. The H_2_O_2_ (1 mM) control was also conducted as a positive control.

### Superoxide dismutase activity

SOD (superoxide dismutase) Colorimetric Activity Kit (EIASODC, Thermo Fisher Scientific, USA) was used to assess the activity of superoxide dismutase in the control and optimised culture. The cultures were centrifuged (6000 rpm, 15 min) after 24 h and washed twice with a sterile PBS buffer. The cells were disrupted using ultrasounds (3 min of 10/10 sec, 130 W, 20 kHz, on ice), centrifuged (10,000 rpm, 2 min), and supernatants were subjected to the colourimetric measurement in a microplate reader.

### Gene expression

Real-time RT-PCR analyses were conducted to monitor the expression of the chosen genes, i.e., pyocyanin production (*phzM*,* phzS*), oxidative stress (s*odB*,* katA*,* katB*,* gpx1*), and efflux pump activity associated with Zn^2+^ ions (*czcA*,* czcD*). The primers were designed in PRIMER3 software based on the *P. aeruginosa* ATCC 27,853 complete genome and are listed in Supplementary materials in Table S2. The RNA extraction from three separate cultures was done using a Total RNA Mini Kit (A&A Biotechnology, Poland). Next, reverse transcription to cDNA was performed with RevertAid RT Kit (ThermoScientific, USA) according to the user’s manual. qPCRs employed SsoAdvanced Universal SYBR Green Supermix (Bio-Rad) using CFX Connect Real-Time PCR Detection System (Bio-Rad). Before calculations, outlier data was identified and removed using the intra-group interquartile range. Analysis was performed using *R* = 2^−ΔΔCt^ calculation methodology with *rpoD* (RNA polymerase sigma factor RpoD) as a reference gene.

### Statistical analysis

The results were analysed employing a Two-Sample t-test in the case of the comparison of two groups and ANOVA with Tukey’s post-hoc test in the case of three analysed groups. The differences were considered significant at *p* ≤ 0.05, *p* ≤ 0.01 or *p* ≤ 0.001.

## Results and discussion

### Statistical optimisation of the process

**Fig. 2:**
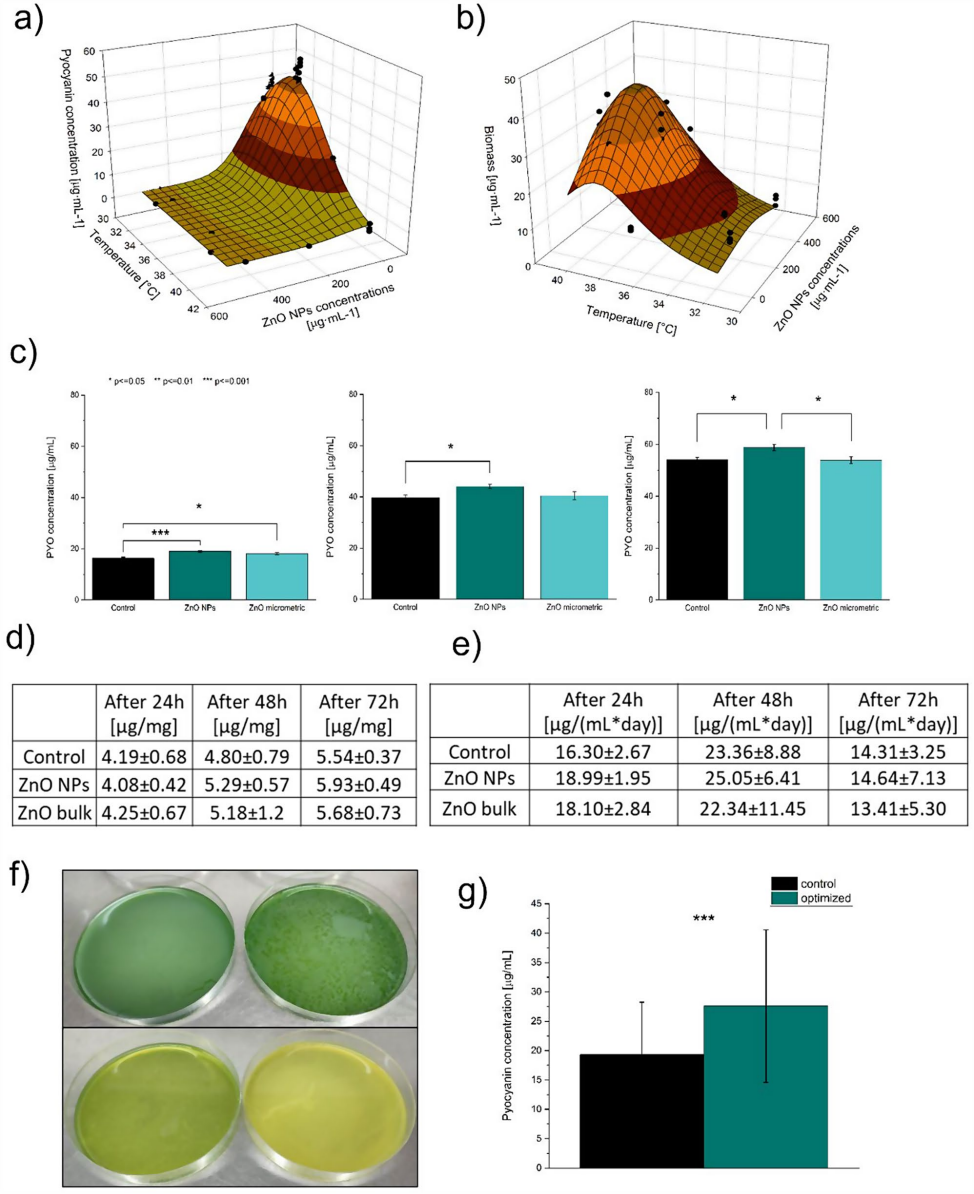
The results of the process optimisation: (**a**) optimised pyocyanin production; (**b**) optimised biomass production, (**c**) pyocyanin production in the control culture, optimised culture with ZnO NPs addition and ZnO bulk addition at different time points, (**d**) substrate/product yield coefficient, (**e**) productivity, (**f**) control and optimised culture for PYO production (cultivated at 32.6 °C, top), control and optimised culture for biomass production (cultivated at 39 °C, bottom); (**g**) the scale-up of pyocyanin production based on the optimisation on the smaller scale

The experiments conducted according to the DoE plan (and some additional ones to obtain a better fit) allowed fitting mathematical functions describing the influence of temperature and ZnO NPs on pyocyanin and biomass production. However, the fit of the surface performed in STATISTICA software resulted in relatively low scores of R^2^_adj_. parameter (Supplementary materials, Fig. S2. and Fig. S3.). For this reason, the surface for pyocyanin production was fitted using a 3D Gaussian equation using Matlab software that allowed a much better fit (Fig. 2a, R^2^_adj_. = 0.95). The calculated optimal ZnO NPs concentration equaled 6.06 µg/mL, while the optimal calculated temperature was 32.6 ℃. Predicted pyocyanin production was equal to 44.16 µg/mL (compared to 39.81 µg/mL according to DoE). In the case of biomass production, the fitted model was 3D Lorentzian. The optimal ZnO NPs content was calculated to 275.75 µg/mL and the temperature to 39 ℃ (R^2^_adj_. = 0.79, Fig. 2b). It is worth underlining that obtained optimal parameters for pyocyanin production were not aligned with biomass production. Pyocyanin was efficiently produced in a lower range of temperatures and with low ZnO NPs addition, while high biomass was obtained while cultivated at higher temperatures and with high ZnO NPs concentrations. Moreover, the observed consistency of the cultures differed, i.e., high ZnO NPs addition induced the formation of a jelly-like structure and high fluorescence of the culture (Fig. 2f bottom, Fig. S3) [32]. Such changes in the consistency of the cultures with high ZnO NPs load may be connected to the production of the polymer, e.g., alginate, and may suggest the induction of the mucoid phenotype as a response to stress [33]. However, this observation was outside the scope of this study and will be further explored in future research. On the other hand, at 32.6 ℃, the control culture and the culture supplemented with 6.06 µg/mL of ZnO NPs produced strong surface biofilm (pellicle) (Fig. 2f top). Nevertheless, the pellicle structure of the optimised culture for pyocyanin production seemed loose and less compact than in the control. The results of the conducted experiments by our research group also underlined the crucial role of the process temperature for efficient production of the pigment and the interactions of the temperature and ZnO NP concentration (Supplementary materials, Fig. S2).

### Yield and productivity calculations

In the next step, we assessed pyocyanin production at three different time points (after 24, 48, and 72 h, Fig. 2c). We compared not only the control and the optimised culture with ZnO NPs but also the samples with 6.06 µg/mL addition of micrometric ZnO. Such an approach allowed the comparison of the effect of different sizes of ZnO on pyocyanin production. The analysis showed that pyocyanin production was higher at all time points in the ZnO NP-supplemented culture than in the control. Moreover, pyocyanin production was also stimulated by the addition of micrometric ZnO after 24 h of incubation. However, at other time points, this effect was not observed. Adding ZnO NPs enhanced pyocyanin production by 16.5% after 24 h, 11.04% after 48 h, and 8.71% after 72 h, on average. Micrometric ZnO also stimulated pyocyanin production by 10.98% after 24 h and 1.97% after 48 h but did not change the production after 72 h. Moreover, the final concentration of pyocyanin obtained from the optimised culture after 72 h (with ZnO NPs) equalled 58.68 µg/mL, which exceeds both predicted values mentioned earlier.

Glycerol quantification (the primary carbon source in King’s A medium) enabled tracking of the use of this substrate and the calculation of the substrate/product yield coefficient and productivity at different time points (Fig. 1d, e.). Glycerol quantification revealed no significant differences between the samples at all time points (Supplementary materials, Fig. S4). Nevertheless, the calculated substrate/product yield coefficient was highest for ZnO NP cultures compared to the control and micrometric ZnO after 48 and 72 h. The productivity generally reached the highest values after 48 h for all tested types of samples. ZnO NPs were characterised by the highest mean values at all time points compared to the control and micrometric ZnO samples and with the stable tendency of the results. Nevertheless, the differences were not statistically significant at *p* ≤ 0.05, possibly due to the high standard deviation between the biological repetitions. The results show that the size of zinc oxide plays a vital role in the stimulative effect on pyocyanin production. On that basis, we also hypothesise that higher pyocyanin production is not related to more efficient use of the carbon source, i.e. glycerol. In that scope, a more probable mechanism under increased pyocyanin production is a shift in the cell’s metabolism.

#### Scale-up of pyocyanin production

The cultures led in 1 L of King’s A medium exhibited significantly higher pyocyanin production in the case of ZnO NPs addition than in the control (Fig. 2g). This shows that the stimulative effect of ZnO NPs is also observed after scaling up the volume circa 66.7 times. Nevertheless, the pyocyanin concentrations obtained in 1 L cultures were significantly lower than in 15 mL (the production reached only 35.77% in the control case and 47.05% in the case of the optimised culture compared to the concentrations obtained in 15 mL). Such a result may be explained by the differences between the two setups, i.e., 15 mL culture was led in a Petri dish where the lid was loosely placed over the culture, whereas 1 L cultures were led in a tray covered with a cling film. It could limit the gas exchange and, therefore, limit pyocyanin production. Also, the ratio of the interphase to the volume is different in these two cases. The calculations of the ratio of interphase to the volume show that they are equal to 4.24 cm^2^/mL for 15 mL cultures and 0.81 cm^2^/mL for 1 L setup. The comparison of these two ratios reveals that the cells in the 15 mL culture have more access to the interphase where gas exchange is taking place (by more than 5-fold). Oxygen availability is a known limiting factor in pyocyanin production [15], so the indicated differences in the interphase area are a probable cause for lower production in bigger volumes.

In our case, the most efficient pyocyanin production occurred at the temperature of 32.6 ℃, which can be considered relatively low, decreasing the potential heating cost of the process. Cardoso et al. [34] named temperature control one of the bioprocess’s most prominent costs. Moreover, our proposed setup does not require agitation, which can be an additional cost. The calculated optimal ZnO NPs content for pyocyanin production is 6.06 µg/mL. Based on the prices of the chemicals used in this research on the producer’s website (sigmaaldrich.com, accessed on 06.02.2024, 112€ for 50 g), the cost of ZnO NPs per litre of the culture is equal to 1.36 euro cents. Such cost could probably be considered negligible due to the stimulative result of 8.71% (in 15 mL cultures) or 42.98% (in the case of scaled-up culture) stimulation of pyocyanin production. Importantly, it should be highlighted that even though the scaled-up production resulted in lower pyocyanin concentration, the stimulation by ZnO NPs was much more pronounced. Moreover, the culturing medium is relatively simple as it consists of only glycerol, peptone, and two inorganic salts. Overall, the proposed setup could be considered as a low-cost-generating method to produce pyocyanin that does not require advanced equipment, stirring, aeration, high process temperature, or costly medium.

#### Growth, viability, and pyoverdine monitoring

**Fig. 3:**
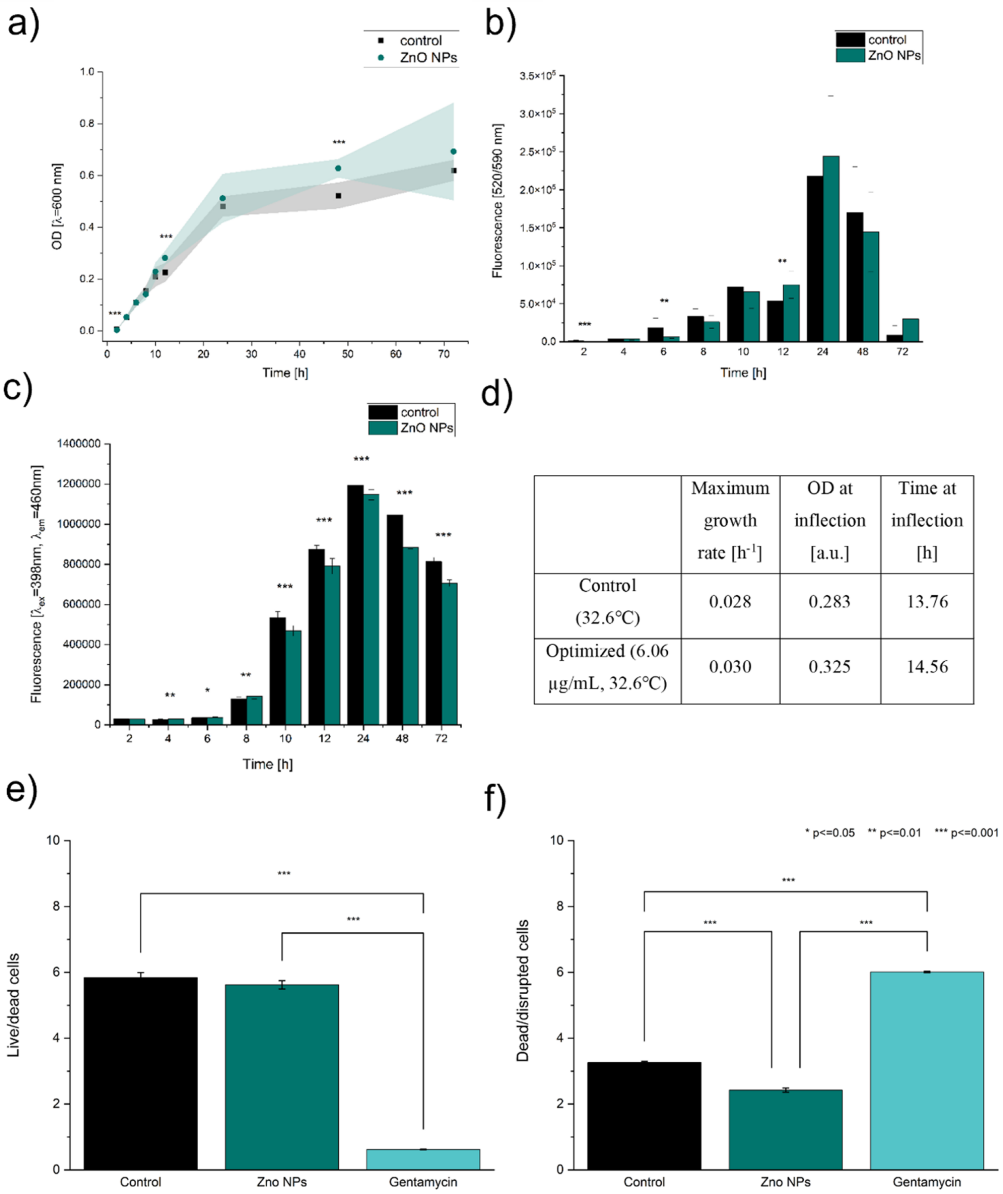
The analyses of the basic physiology of *P. aeruginosa* at optimised conditions: **a**) growth curve, (**b**) viability analysis, (**c**) pyoverdine production, (**d**) calculated growth parameters of the tested and optimised culture, (**e**) cytometric analysis of Live/Dead ratio of the cells, and (**f**) cytometric analysis of Dead/Disrupted ratio of the cells

The significant differences between the OD of the control and the tested culture were recorded at several time points. Higher OD was noted for the control in the 2nd hour of incubation and the optimised culture at 12th and 48th hour of the growth (Fig. 3a). The viability differed at 2nd, 6th hour (higher for the control), and 12th hour (higher for the optimised sample) (Fig. 3b.). These results allow the assumption that overall higher pyocyanin production is probably not the direct effect of higher number of the fast-growing and viable cells. On the other hand, many significant differences were noted during fluorometric monitoring of the pyoverdine production (Fig. 3c.). During the exponential phase of the growth (4th, 6^th,^ and 8th hour), higher pyoverdine production was noted for the optimised culture than for the control. However, at all further time points (i.e. 10th, 12th, 24th, 48^th,^ and 72nd hour), the control culture exhibited higher fluorescence values assigned to pyoverdine production. This signal could not be accounted for pyocyanin because this substance has a lower fluorescence level under tested wavelengths than King’s A broth (Supplementary materials, Fig. S5). Such results may suggest that in the late exponential/early stationary phase, there is a particular switch in production due to the presence of ZnO NPs.

The obtained OD data allowed the fitting of the logistic function (R^2^_adj_. = 0.97 for both control and ZnO NP culture, Fig. S6.) that was used to calculate the growth parameters. The growth rate of the optimised culture was slightly higher than in the control culture (Fig. 3d.). Moreover, the inflexion time of the control culture was reached faster (after 13.76 h) than in the optimised culture (14.56 h). The inflexion point is an indicator of the beginning of the growth rate decline in the culture [35]. The difference between the inflexion time (48 min) could suggest that the cells incubated with ZnO NPs maintained a higher growth rate for longer. Nevertheless, such an effect may also be connected to the production of the pigments that interfere with OD measurements (Supplementary materials, Fig. S7.) [36, 37].

Viability assay (LIVE/DEAD staining) using flow cytometry showed that the ratio of viable/dead cells did not differ significantly between the control and ZnO NPs samples after 24 hours of incubation (Fig. 3e.). Therefore, the observed rise in pyocyanin production was not linked to the changes in the viability of the bacterial population. However, the ratio of dead to disrupted cells was significantly higher for the control than for the ZnO NP samples (Fig. 3f.). This shows that the number of cells with disrupted cell membranes that are stained with both green and red dye (also called ‘intermediate state’ cells [28, 38]) is higher in the case of ZnO NPs presence and may indicate that the cells are under stress. Easier identification of the dead population of bacteria in cytograms and subpopulations characterised by disrupted membrane was possible thanks to the additional control with gentamycin (Supplementary materials, Fig. S9).

Moreover, we Zhu et al. [39] describing that elevated pyocyanin production can be linked to lower susceptibility to antibiotics. Production of pyocyanin is associated with redox cycling, which enables the oxidation of glucose and pyruvate into acetate, which is later used to produce ATP. It allows the bacterium to generate proton-motive force and maintain redox homeostasis [40]. The effect is strengthened by introducing oxidising potential and can be reversed by introducing a reducing potential to the bacterial population, disrupting redox cycling and increasing susceptibility to antibiotics [41]. Our results confirmed that the inhibition zone of *P. aeruginosa* exposed to a stimulative concentration of ZnO NPs for 6 h was smaller than in the control culture for some of the tested antibiotics, especially piperacillin combined with tazobactam (Supplementary materials, Fig. S10). This finding raises concerns as ZnO NPs are incorporated into many commercial products and will ultimately enter the environment. As *P. aeruginosa* is present in many habitats, e.g., water, it can be exposed to sublethal ZnO NPs and perhaps become less susceptible to antibiotics. Similar concerns were voiced by Ouyang et al. [42] who showed the concentration-dependent influence of ZnO NPs on *Pseudomonas putida* and suggested that long-term exposure to low concentrations of ZnO NPs can result in undesired effects, e.g., resistance to antibiotics or increased biofilm formation. However, the proposed set up for pyocyanin production is strictly limited to laboratory environment and should not impact the antibiotic resistance in the environment.

#### Biofilm and eDNA studies

**Fig. 4:**
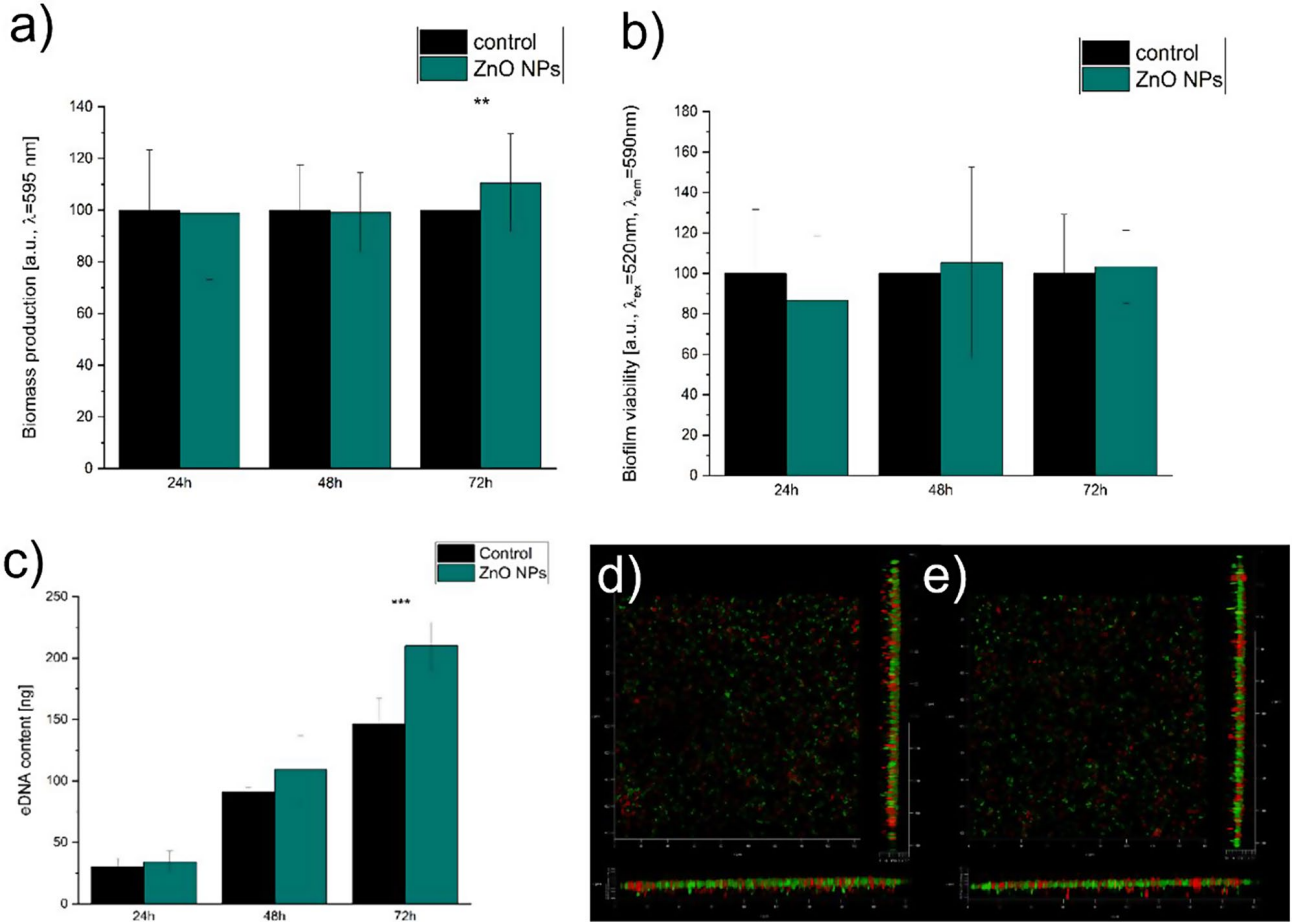
The analyses of the biofilm characteristics: (**a**) biofilm biomass, (**b**) viability of the biofilm, (**c**) eDNA, (**d**) the biofilm of the control culture (CLSM image), and (**e**) the biofilm of the ZnO NPs-supplemented culture (confocal laser scanning microscopy)

The analysis of the biofilm biomass of the control and ZnO NPs-supplemented cultures revealed only one significant difference. The biomass in the ZnO NPs sample was significantly higher than the control after 72 h (Fig. 4a). At the same time, the viability of the biofilm (in comparison to the control) did not significantly change between these samples (Fig. 4b.). More changes were observed for micrometric ZnO as the viability was significantly lower than in control after 24 and 72 h and the biomass was lower than in control after 48 h (Supplementary materials, Fig. S11). Nevertheless, due to the pronounced production of the biofilm in the form of a pellicle at the gas-liquid interphase (Fig. 2f.), biofilm analysis may show limited accuracy as it requires washing steps in wells that, at least partially, remove the pellicle. Similarities in viability were also detected in biofilms and indicated limited toxicity of ZnO applied at low (optimised) concentrations. Confocal microscopy confirmed no pronounced differences in 24-hour-old biofilm (Fig. 4d, e.). Furthermore, we noted a slightly higher number of viable cells in the sample with ZnO NPs (Supplementary materials, Fig. S12).

The extracellular DNA analysis revealed significantly increased eDNA content in the sample with ZnO NP after 72 h (in comparison to the control, Fig. 4c). Nevertheless, the trend of the higher mean values of eDNA content in ZnO NPs samples than in the control were consistently noted at all tested timepoints. The same observation was actual for micrometric ZnO (Supplementary materials, Fig. S13). Therefore, we confirmed that the size of ZnO did not alter the upregulated eDNA concentration; it consistently enhanced the production of eDNA. These results match the findings of Das et al. [27], who suggested that increased pyocyanin production is intertwined with higher eDNA levels. They suggested that, in their case, the production of H_2_O_2_ was triggering this phenomenon.

Colony morphology analysis did not reveal pronounced differences between the control and ZnO NPs-supplemented culture (Supplementary materials, Fig. S14). Nevertheless, in both cases, we observed plaques indicating the probable cell lysis in these areas, suggesting that at the tested culture conditions, the prophage induction can be observed. This is expected after considering that the genome of *P. aeruginosa* ATCC 27,853 contains sequences of 7 prophages [43, 44].

### Analyses of cellular stress

**Fig. 5:**
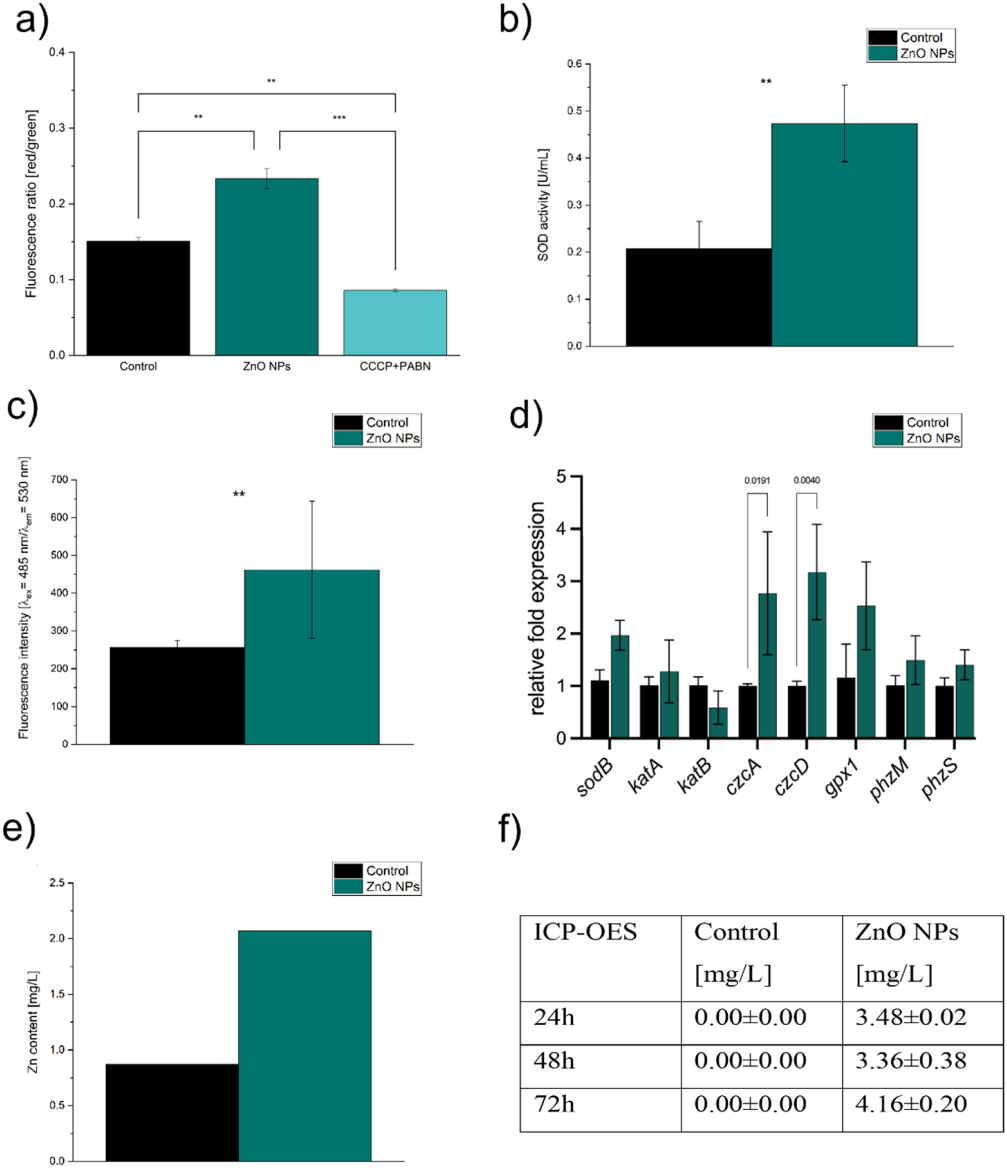
The analyses of the cellular stress: (**a**) membrane potential of the cells, (**b**) SOD activity in the tested and optimised culture; (**c**) general ROS (DCFH-DA assay), (**d**) gene expression, (**e**) zinc content in bacterial biomass (AAS), (**f**) zinc content in culture filtrates at different timepoints (ICP-OES)

Membrane potential assay revealed an increased membrane potential (in comparison to the control, Fig. 5a.) in the culture incubated with ZnO NPs. Other authors who recorded the increased red-to-green ratio in such an assay concluded that it indicates adaptive resistance [45, 46]. Superoxide dismutase activity was upregulated in the optimised culture after 24 h of incubation (Fig. 5b.). This may indicate the generation of oxidative stress by ZnO NPs added to the culture. However, *P. aeruginosa* can also naturally exhibit higher SOD activity due to pyocyanin production that becomes more pronounced in the late log phase [47]. Therefore, we also tested if oxidative stress is present before pyocyanin production starts, i.e., during the logarithmic growth phase. CellROX Green assay allowed for the observation of a slightly higher number of events after 6 h of incubation, which indicated the presence of superoxide radicals in the samples (Fig. S15). However, the differences were not statistically significant. McBee et al. [48] reported that this assay enables superoxide radical detection. Therefore, taking the obtained results into account, we conclude that the level of this radical might have slightly increased but did not significantly change after ZnO supplementation. Nevertheless, we confirmed using the DCFH-DA assay that general ROS levels increase after 12 h of incubation (Fig. 5e.). Additional pyocyanin quantitation at this time point excluded interference between the metabolite and DCFH-DA. In summary, the results showed a significant increase of ROS in culture supplemented with ZnO NPs; thus, *P. aeruginosa* was exposed to oxidative stress during the process.

### Gene expression

The results of gene expression analysis are depicted in Fig. 5f. Almost all tested genes were characterised with higher relative fold expression in the case of ZnO NPs supplementation than in the control. The only exception was *katB*, which encodes hydrogen peroxide-inducible catalase and exhibited lower expression than the control. This result may confirm that oxidative stress was not a result of H_2_O_2_ and an additional indication that increased concentration of eDNA (see Biofilm and eDNA studies) had another trigger [30]. Genes *phzS* and *phzM* had higher expression than the control, which is expected for higher pyocyanin production. These genes transform phenazine-1-carboxylic acid (PCA) by encoding putative phenazine-specific methyltransferase and flavin-containing monooxygenase [49]. The tested antioxidant enzymes (besides *katB*) exhibited higher expression in the ZnO-supplemented samples than the control. This result was a consequence of oxidative stress. However, it is unclear whether the stress was related to the activity of ZnO NPs or a result of higher pyocyanin production. The most pronounced rise in the expression of the genes was noted for the genes encoding metal cation efflux transporters, i.e. *czcA* and *czcD*. The first one encodes a protein that is a part of the RND (Resistance-Nodulation-Division) efflux CzcCBA pump, facilitating the removal of the excess zinc ions from the cytoplasm to the extracellular environment [50]. In contrast, the *czcD* gene encodes the protein that constitutes the Cation Diffusion Facilitator (CDF), an efflux system in *P. aeruginosa* responsible for removing zinc ions to periplasmic space. However, *czcD* is not essential in zinc resistance and is constitutively expressed after adding zinc. Based on these results, we hypothesise that zinc penetrated the cells, and the cells actively tried to remove the excess zinc using both studied pumps, RND and CDF [50].

A rising number of articles showing the stimulative effect of sublethal concentrations of nanoparticles (or other antimicrobial compounds such as royal jelly) on virulence factors of bacteria has emerged during the last decade [51–57]. Most works show such an effect of Ag NPs. Among the reported effects of Ag NPs on *P. aeruginosa* are the upregulation of quorum sensing and efflux pump activity, enhanced biofilm formation, lipopolysaccharide biosynthesis, swarming and twitching motility, and the increased production of elastase and pyocyanin. Other authors [57] showed the stimulative effect of sublethal concentrations of nanoceria on pyocyanin, pyoverdine and alginate production. In addition, the analysis of the *P. aeruginosa* proteome revealed the upregulation of the proteins associated with metabolic pathways, biosynthesis of amino acids, microbial metabolism in diverse environments, redox homeostasis and oxidative stress. Some authors also underlined that the response of *P. aeruginosa* to nanoparticles can be strain-dependent [51, 52, 58]. These effects are not restricted to pseudomonads, and the stimulation with a sublethal concentration of ZnO NPs was noted in biofilm and endospore formation by *Bacillus cereus* in response to stress [53]. In this work, we show that two different dosages of sublethal concentrations of ZnO NPs combined with different process temperatures led to adverse results – in one case, the stimulation of pigment production and, in the other, the abolishment of pyocyanin production and mucoid phenotype induction. These findings point towards testing a wide range of sublethal concentrations of NPs as many interesting and potentially new observations can occur.

## Zinc content

ICP-OES analysis allowed quantification of zinc in the culture filtrates (Fig. 5f). The zinc levels were below the detection limit in each control sample. However, the level of zinc in the pure King’s A medium was quantified with this method and equalled 0.09 mg/L. It shows that the culture probably used most of the available zinc for the metabolic processes during the first 24 h of the culture. In the optimised culture, the zinc content equalled 3.48, 3.36, and 4.16 mg/L after 24, 48 and 72 h, respectively. On the other hand, the zinc concentration in filtrates from micrometric ZnO cultures showed an overall lower Zn content than in the case of ZnO NPs (Supplementary materials, Fig. S16).

AAS analysis revealed higher levels of zinc in the biomass incubated with ZnO NPs than in the control (Fig. 5e.). This may suggest that zinc was partially uptaken by the cells or was permanently attached to their surface. On the other hand, the observed upregulation of genes coding efflux pumps suggests that zinc could be accumulated in the biomass, particularly the periplasmic space where it was transported by the CDF pump, or sequestrated outside the cell by metallophores after removal with the RND pump [50, 59].

## Conclusions

We showed that ZnO NPs can have ambiguous effects on pyocyanin production, i.e., intensifying it at low concentrations or abolishing it while added in excess, simultaneously forcing cells to induce the mucoid phenotype and produce exopolymeric material. Higher pyocyanin production does not seem to be intertwined with faster growth or significant changes in the viability of the cells. Moreover, the size of the ZnO used influences the outcome. ZnO nanoparticles act differently than micrometric ZnO, as only the nanoform consistently stimulates pyocyanin production. Our findings suggest that the observed phenomenon results from cellular stress, i.e. oxidative stress, increased membrane potential, and zinc accumulation inside the cell and in the biomass. Moreover, it was confirmed that the stimulative effect of ZnO NPs on pyocyanin production also occurs in a scaled-up culture, resulting in smaller concentrations but significantly higher stimulation rate, which is a good prospect for enhanced production on a large scale. The presented approach shows that sublethal cellular stress levels can be harnessed and optimised to produce the selected metabolite more efficiently.

### Electronic supplementary material

Below is the link to the electronic supplementary material.

Supplementary Material 1

## Data Availability

The data generated in this research is available in Bridge of Knowledge data repository under DOI: 10.34808/4j3e-dx43.
