# Supplementary material for: Optimised stress – intensification of pyocyanin production with zinc oxide nanoparticles
Source: Microb Cell Fact. 2024 Jul 27;23:215. doi: 10.1186/s12934-024-02486-y (PMC11282796; doi:10.1186/s12934-024-02486-y)
Supplement: Supplementary file 1 — Supplementary Material 1 [file 12934_2024_2486_MOESM1_ESM.pdf]

Supplementary materials

Optimized stress – intensification of pyocyanin production with zinc oxide nanoparticles

Joanna Honselmann genannt Humme[1*], Kamila Dubrowska[1], Bartłomiej Grygorcewicz[2], Marta Gliźniewicz[2], Oliwia Paszkiewicz[1,3], Anna Głowacka[3], Daniel Musik[4], Grzegorz Story[1], Rafał Rakoczy[1,5], Adrian Augustyniak[1, 6]

[1] West Pomeranian University of Technology in Szczecin, Faculty of Chemical Technology and Engineering, Department of Chemical and Process Engineering, Piastów Avenue 42, 71-065 Szczecin, Poland

[2] Pomeranian Medical University in Szczecin, Faculty of Medicine and Dentistry, Powstańców Wielkopolskich 72, 70-111 Szczecin

[3] West Pomeranian University of Technology in Szczecin, Department of Environmental Engineering, Piastów Avenue 50a, Szczecin, 70-311, Poland

[4] ESC Global Sp. z o.o., Słoneczny Sad 4F, 72-002 Dołuje, Poland

[5] Center for Advanced Materials and Manufacturing Process Engineering (CAMMPE), Piastow Avenue 42, 71-065 Szczecin, Poland

[6] Technische Universität Berlin, Chair of Building Materials and Construction Chemistry, Gustav-Meyer-Allee 25, 13355 Berlin, Germany

S1. Materials and methods

S1.1. *Statistical optimization of the process*

The experiments were conducted according to the plan created in STATISTICA software presented in Tab. S1.

Tab. S1. Plan of experiments

No. of experiment	Repetitions	Concentration of ZnO NPs [µg/mL]	Temperature [°C]
2	1	100.00	39.00
8	1	225.00	40.77
18	2	225.00	40.77
28	3	225.00	40.77
10 (C)	1	225.00	37.00
4	1	500.00	39.00
13	2	500.00	32.00
11	2	100.00	32.00
20 (C)	2	225.00	37.00
24	3	500.00	39.00
30 (C)	3	225.00	37.00
7	1	225.00	33.23
21	3	100.00	32.00
25	3	9.38	37.00
15	2	9.38	37.00
1	1	100.00	32.00
3	1	500.00	32.00
29 (C)	3	225.00	37.00

26	3	440.62	37.00
17	2	225.00	33.23
16	2	440.62	37.00
22	3	100.00	39.00
9 (C)	1	225.00	37.00
14	2	500.00	39.00
23	3	500.00	32.00
5	1	9.38	37.00
27	3	225.00	33.23
19 (C)	2	225.00	37.00
6	1	440.62	37.00
12	2	100.00	39.00

S1.2. *Pyocyanin quantification*

Pyocyanin concentration was quantified by calculation from the calibration curve (Fig. S1). The measurements were conducted at 520 nm in the volume of 100 µL using a microplate reader.

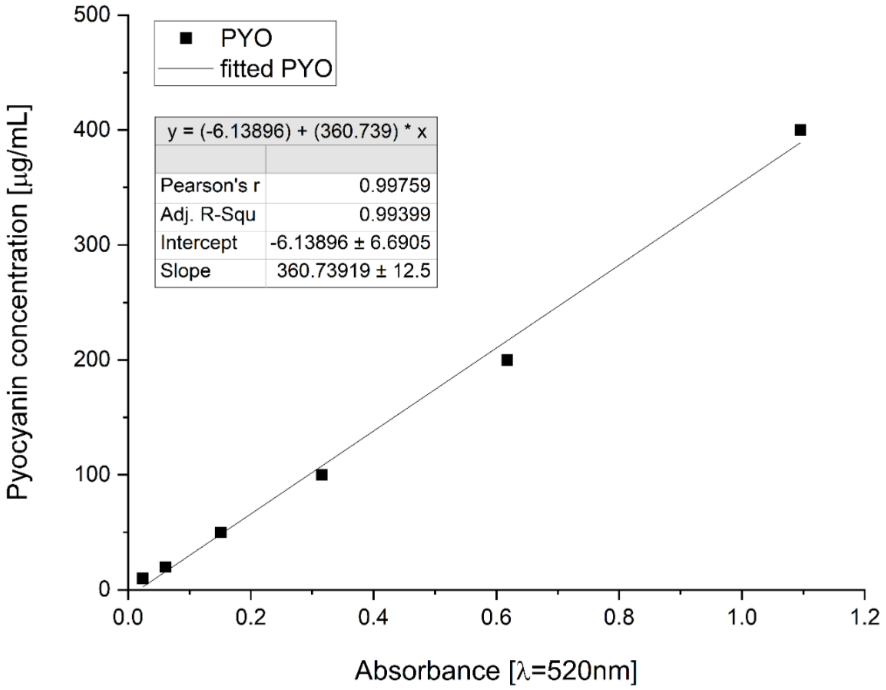

Fig. S1. Calibration curve of pyocyanin dissolved in 0.2N HCl measured spectrophotometrically.

S1.3. Antibiotic susceptibility

Antibiotic susceptibility was tested according to EUCAST recommendations for disk diffusion test with modified incubation temperature, that was calculated as optimal for the pyocyanin production process (32.6°C). Tested antibiotics covered ciprofloxacin (5 mg/L), tobramycin (10 mg/L), piperacillin + tazobactam (30 mg/L + 6 mg/L), cefepime (30 mg/L) and imipenem (10 mg/L). After the incubation time, the inhibition zones were measured and compared.

S1.4. Colony morphology

The morphology of the colony (also called 'colony biofilm') was observed on King's A agar supplemented with 20 μg/mL of Coomassie Blue and 40 μg/mL of Congo Red (Dietrich et al. 2013 with modifications). Shortly, 5 μL of 6-hour-old culture (control or ZnO NPs-supplemented) was spotted on the surface of the medium and incubated for 6 days at 25°C and 32.6°C.

S1.5. Oxidative stress

To investigate if reactive oxygen species were generated in the ZnO NPs culture, the flow cytometry assay for detection of superoxide radical ($O_2^{-\cdot}$) in the culture was carried out (CellROX Green, Thermo Fisher Scientific, USA). Shortly, the cultures were led for 6h (mid-log phase where no pyocyanin is being produced), diluted, and incubated with ROS generator, i.e. menadione (100 μg/mL in DMSO) and TBHP (200 μM), for 30 minutes. Then, the staining was performed with CellROX Green for 15 minutes. Data was collected on BD Accuri™ C6 Plus. Ten thousand events were recorded. Analyses were performed on FCS Express (De Novo Software, Pasadena, CA, USA). Mean fluorescence intensity (MFI) was calculated on green channel and compared between samples.

S1.6. Gene expression

The sequences of the genes selected for RT-qPCR analysis were created in PRIMER3 software based on *P. aeruginosa* ATCC 27853 reference genome (https://genomes.atcc.org/genomes/a96dd3fec62a4470) and are shown in Tab. S2.

Tab. S2. Sequences of the primers used in the study.

	Name	Gene	Primer F	Primer R	Product length (bp)
1	Phenazine-specific methyltransferase	*phzM*	CGAATTGACCAAGGC CATCC	AGCAGGTAGA TATCGCCGTT	180
2	Phenazine-1-carboxylate N-methyltransferase	*phzS*	CGTCGGCATCAATAT CCAGC	GATGGATCGA GTACTGCGGA	185
3	Catalase A	*katA*	GCTTCTGGGTCAAGT TCCAC	CTGCACGTAC ATCTTCCAGC	161
4	Catalase B	*katB*	ACCCTGCTGTATTCC AACGA	TCGGATCGAG GTTCTTCTGG	154
5	Superoxide dismutase	*sodB*	TGCCTTACGAAAAGA ACGCC	GGAGGAGCTC TTGACGATCT	161
6	Glutathione peroxidase	*gpx1*	CCATCAAGGGCGAAC AGAAG	AAGCTCACCC CGTAGTTCAG	238
7	Metal cation efflux system protein	*czcD*	CAACAGCCTGAACGT CAAGG	AGGACCCAGA AACCGATCAG	150
8	Metal cation efflux system protein	*czcA*	CGATGACATGGAGCA ACTGG	GATTGCCACC GTTTGCTGTA	187
9	Housekeeping gene (RNA polymerase sigma factor)	*rpoD*	CGAGGAAGAAGAAA GCGACG	CAGCTTGATC GGCATGAACA	205

S2. Results

S2.1. Statistical optimization of the process

The results obtained by the DoE approach were analyzed in STATISTICA software. It allowed the fitting of the quadratic model with interactions and obtained the value of $R^2_{adj.}$ = 0.88. All the tested factors (both linear and quadratic) and their interactions significantly influenced pyocyanin production (as shown on the Pareto chart). The results also allowed the attempt to calculate the optimal conditions for pyocyanin production, i.e., obtained values were 5 µg/mL of ZnO NPs and 30°C. However, based on the experimental results, we observed that these values may not be fully accurate. Therefore, we decided to attempt fitting a surface described by another equation to obtain optimal values that are true in comparison with empirical data. The best fit was obtained using 3D Gaussian fit (R^2 adj. = 0.95) and allowed calculation of the optimal conditions.

Similarly, as in the case of pyocyanin production, the DoE approach in STATISTICA software for biomass optimization resulted in a quite low fit ($R^2_{adj.}$ = 0.69). According to this fit the factors that significantly influenced biomass production were temperature (linear) and ZnO NPs concentration (quadratic). This fit resulted in the calculation of optimal conditions equal to, i.e. obtained values were 254.69 µg/mL of ZnO NPs and 40.77°C. Just like in the case of pyocyanin, based on the experimental results, we observed that these values are not accurate. Therefore, we attempted fitting a 3D Lorentzian fit (R^2 adj. = 0.79) that was characterized by better fit parameters and allowed calculating the optimal conditions close to the empirical results.

Factor	Effect Estimates; Var.:Pyocyanin; R-sqr=,88765; Adj:,87985 (Spreadsheet2)<br>2 factors, 1 Blocks, 78 Runs; MS Residual=47,39135<br>DV: Pyocyanin Effect	Std.Err.	t(72)	p	-95,% Cnf.Limt	+95,% Cnf.Limt	Coeff.	Std.Err. Coeff.	-95,% Cnf.Limt	+95,% Cnf.Limt
Mean/Interc.	6,0669	1,996618	3,0386	0,003311	2,0867	10,0471	6,0669	1,996618	2,0867	10,0471
(1)ZnO NPs(L)	-26,4811	2,375317	-11,1484	0,000000	-31,2162	-21,7460	-13,2405	1,187659	-15,6081	-10,8730
ZnO NPs(Q)	29,3661	5,074920	5,7865	0,000000	19,2495	39,4828	14,6831	2,537460	9,6247	19,7414
(2)Temperature(L)	-14,4257	2,681152	-5,3804	0,000001	-19,7705	-9,0809	-7,2129	1,340576	-9,8853	-4,5405
Temperature(Q)	-21,5084	4,446859	-4,8368	0,000007	-30,3730	-12,6437	-10,7542	2,223430	-15,1865	-6,3219
1L by 2L	19,9764	3,231971	6,1809	0,000000	13,5336	26,4193	9,9882	1,615986	6,7668	13,2096

Factor	ANOVA; Var.:Pyocyanin; R-sqr=,88765; Adj:,87985 (Spreadsheet2)<br>2 factors, 1 Blocks, 78 Runs; MS Residual=47,39135<br>DV: Pyocyanin SS	df	MS	F	p
(1)ZnO NPs(L)	5890,15	1	5890,151	124,2875	0,000000
ZnO NPs(Q)	1586,85	1	1586,845	33,4839	0,000000
(2)Temperature(L)	1371,93	1	1371,929	28,9489	0,000001
Temperature(Q)	1108,68	1	1108,685	23,3942	0,000007
1L by 2L	1810,51	1	1810,507	38,2033	0,000000
Error	3412,18	72	47,391		
Total SS	30371,23	77			

Fitted Surface; Variable: Pyocyanin
2 factors, 1 Blocks, 78 Runs; MS Residual=47,39135
DV: Pyocyanin

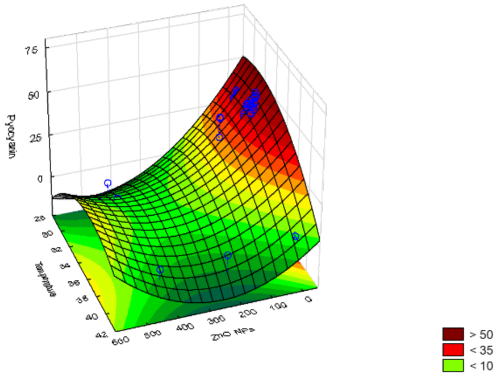

Pareto Chart of Standardized Effects; Variable: Pyocyanin
2 factors, 1 Blocks, 78 Runs; MS Residual=47,39135
DV: Pyocyanin

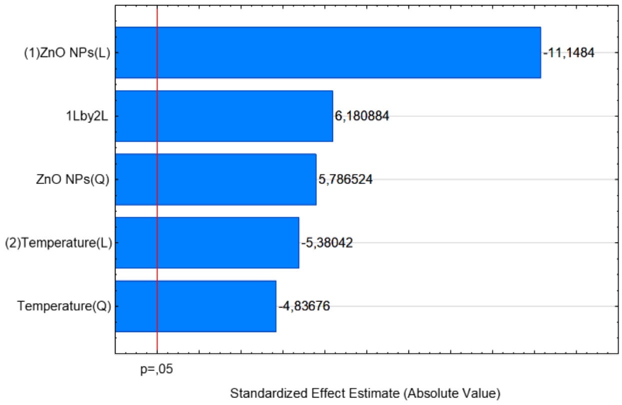

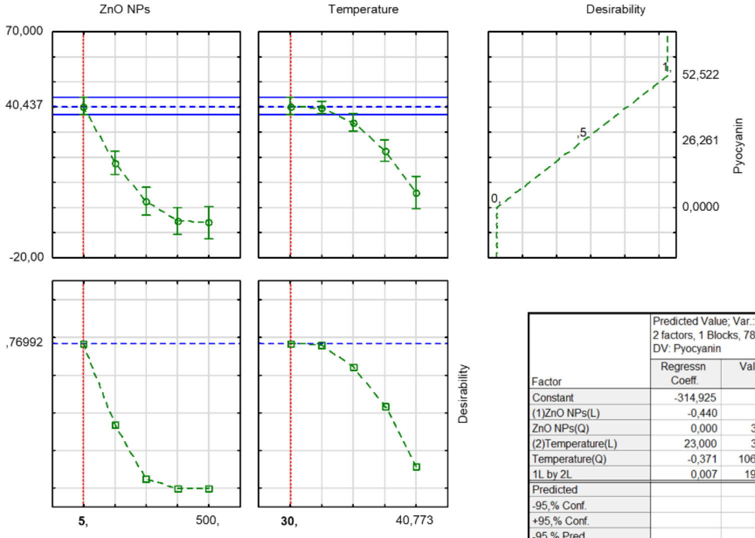

Factor	Predicted Value; Var.:Pyocyanin; R-sqr=,88765; Adj:,87985 (Spreadsheet2)<br>2 factors, 1 Blocks, 78 Runs; MS Residual=47,39135<br>DV: Pyocyanin Regressn Coeff.	Value	Coeff. * Value
Constant	-314,925		
(1)ZnO NPs(L)	-0,440	6,060	-2,664
ZnO NPs(Q)	0,000	36,724	0,009
(2)Temperature(L)	23,000	32,600	749,797
Temperature(Q)	-0,371	1062,760	-393,890
1L by 2L	0,007	197,556	1,480
Predicted			39,807
-95,% Conf.			37,355
+95,% Conf.			42,258
-95,% Pred.			25,866
+95,% Pred.			53,747

Fig. S2. DoE optimization of pyocyanin production.

Factor	Effect	Std.Err.	t(24)	p	-95,% Cnf.Limt	+95,% Cnf.Limt	Coeff.	Std.Err. Coeff.	-95,% Cnf.Limt	+95,% Cnf.Limt
Mean/Interc.	26,08495	2,069779	12,60277	0,000000	21,8131	30,35676	26,08495	2,069779	21,81313	30,35676
(1)ZnO NPs(L)	1,83301	2,189344	0,83724	0,410716	-2,6856	6,35160	0,91651	1,094672	-1,34278	3,17580
ZnO NPs(Q)	-8,89125	2,615478	-3,39947	0,002361	-14,2893	-3,49317	-4,44562	1,307739	-7,14466	-1,74658
(2)Temperature(L)	14,49185	2,118692	6,84000	0,000000	10,1191	18,86462	7,24593	1,059346	5,05954	9,43231
Temperature(Q)	-3,00731	2,373772	-1,26689	0,217352	-7,9065	1,89192	-1,50365	1,186886	-3,95327	0,94596
1L by 2L	-0,41996	2,193928	-0,19142	0,849808	-4,9480	4,10809	-0,20998	1,096964	-2,47400	2,05404

Effect Estimates; Var.:Biomass; R-sqr=,74588; Adj:,69294 (Spreadsheet1)
2 factors, 1 Blocks, 30 Runs; MS Residual=31,99245
DV: Biomass

Factor	SS	df	MS	F	p
(1)ZnO NPs(L)	22,426	1	22,426	0,70098	0,410716
ZnO NPs(Q)	369,718	1	369,718	11,55642	0,002361
(2)Temperature(L)	1496,786	1	1496,786	46,78559	0,000000
Temperature(Q)	51,348	1	51,348	1,60501	0,217352
1L by 2L	1,172	1	1,172	0,03664	0,849808
Error	767,819	24	31,992		
Total SS	3021,471	29			

ANOVA; Var.:Biomass; R-sqr=,74588; Adj:,69294 (Spreadsheet1)
2 factors, 1 Blocks, 30 Runs; MS Residual=31,99245
DV: Biomass

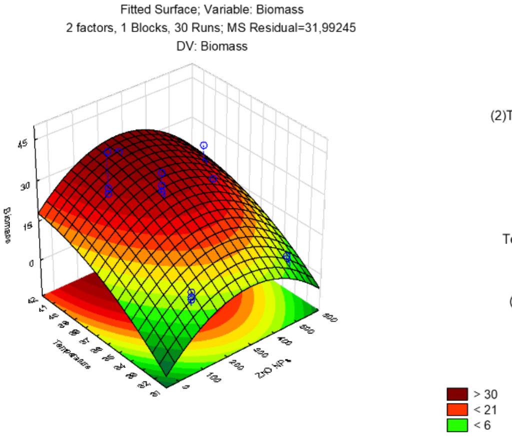

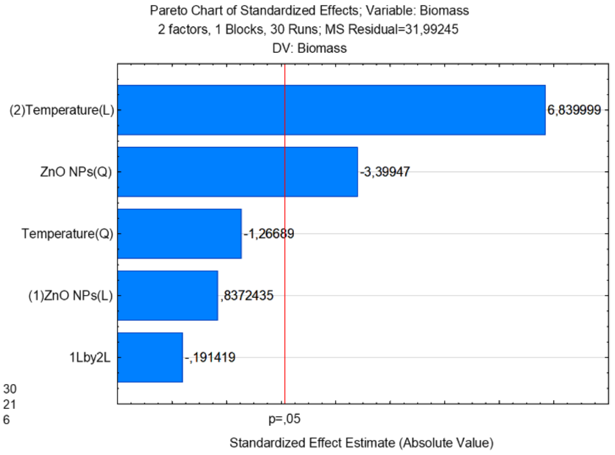

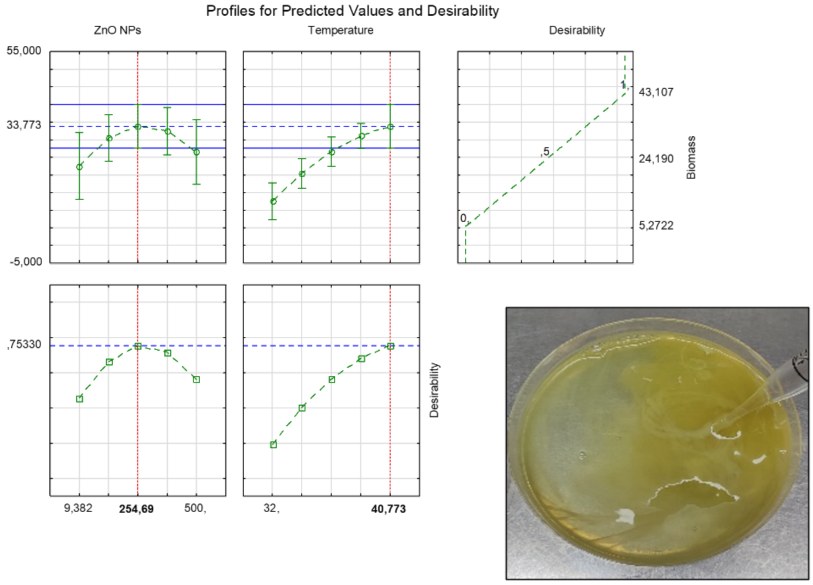

Fig. S3. DoE optimization of biomass production and the jelly-like structure of the optimized biomass production after mixing of the culture.

Pyocyanin yield and productivity calculations

Glycerol concentration was quantified in the control, ZnO NPs-supplemented, and micrometric ZnO-supplemented culture. The statistical analysis of the glycerol content revealed no significant differences between the samples. This suggests that elevated pyocyanin production is most probably not a result of more effective use of the main carbon source.

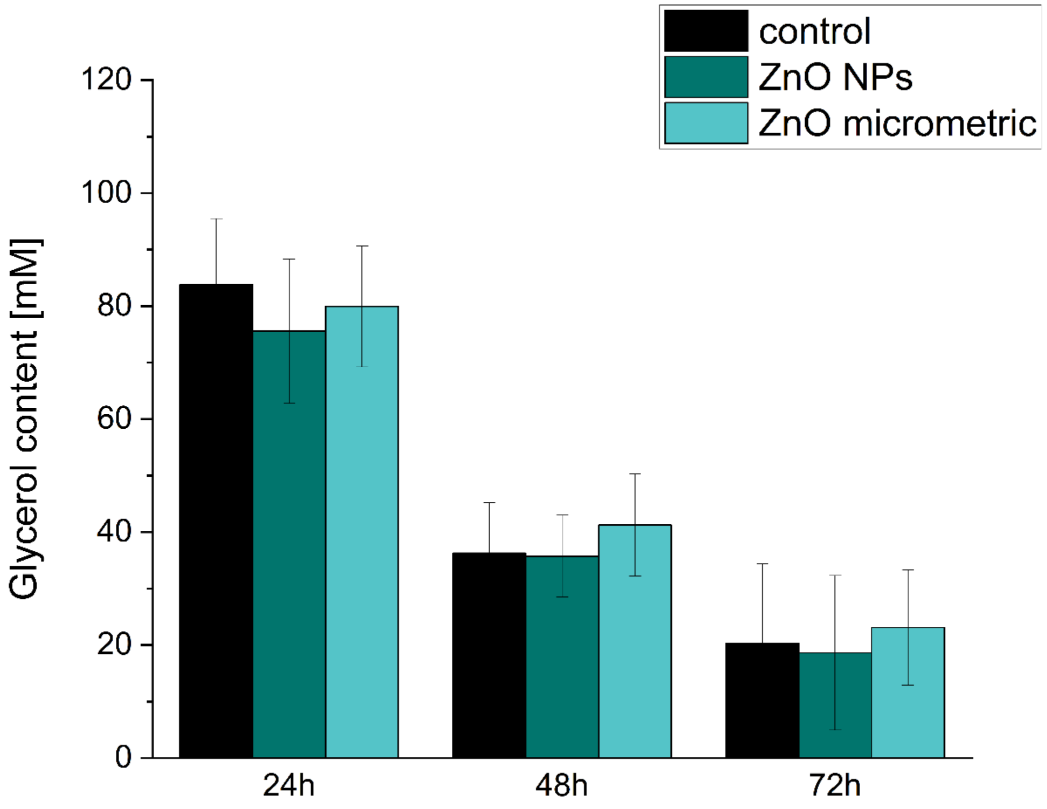

Fig. S4. Glycerol quantification in filtered cultures.

S2.2. Growth, viability, and pyoverdine monitoring

The fluorescence scan was performed (Fig. S5) to verify if the measured fluorescence could be assigned to pyocyanin. Nevertheless, the signal from pyocyanin dissolved in the medium is lower than signal of the medium itself.

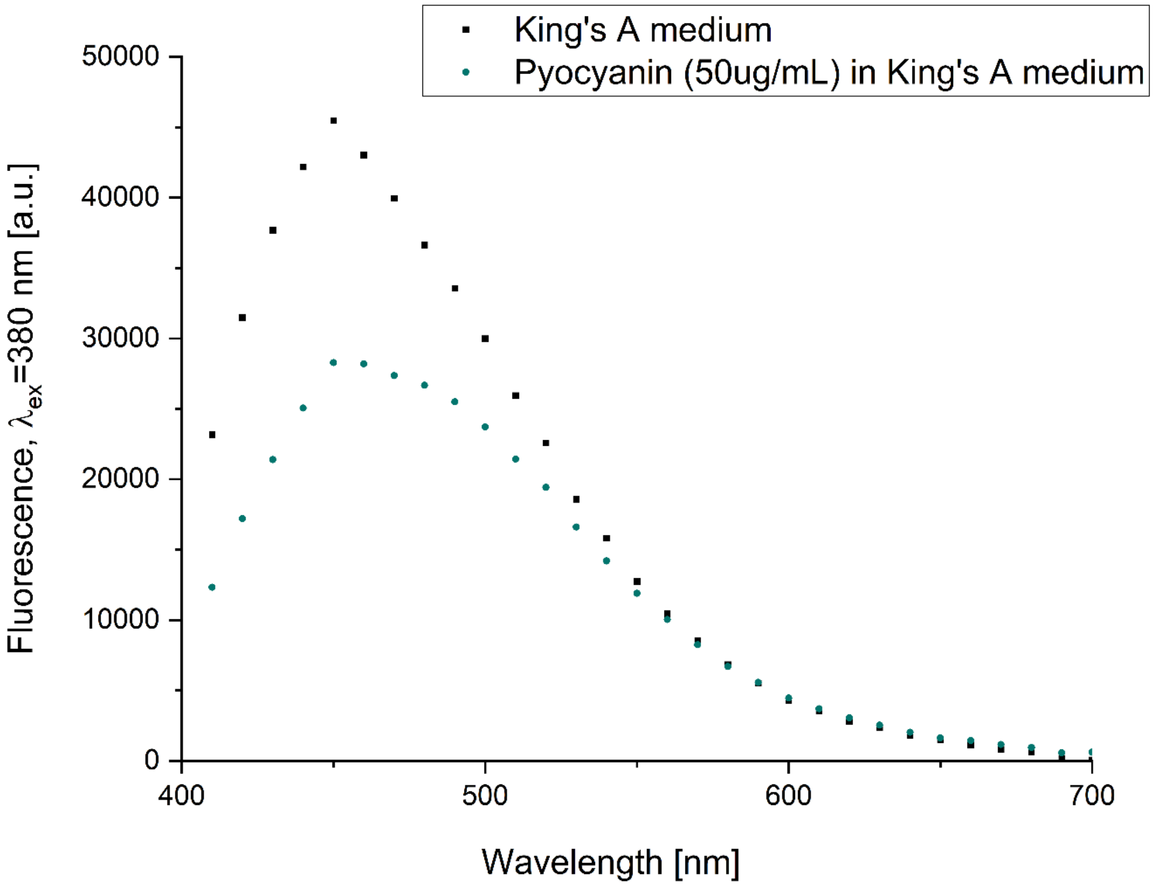

Fig. S5. Fluorescence spectrum of pure King's A medium and pyocyanin dissolved in King's A medium.

The obtained OD data was used to fit the logistic function (Fig. S5.) and to calculate the growth rate and inflection point.

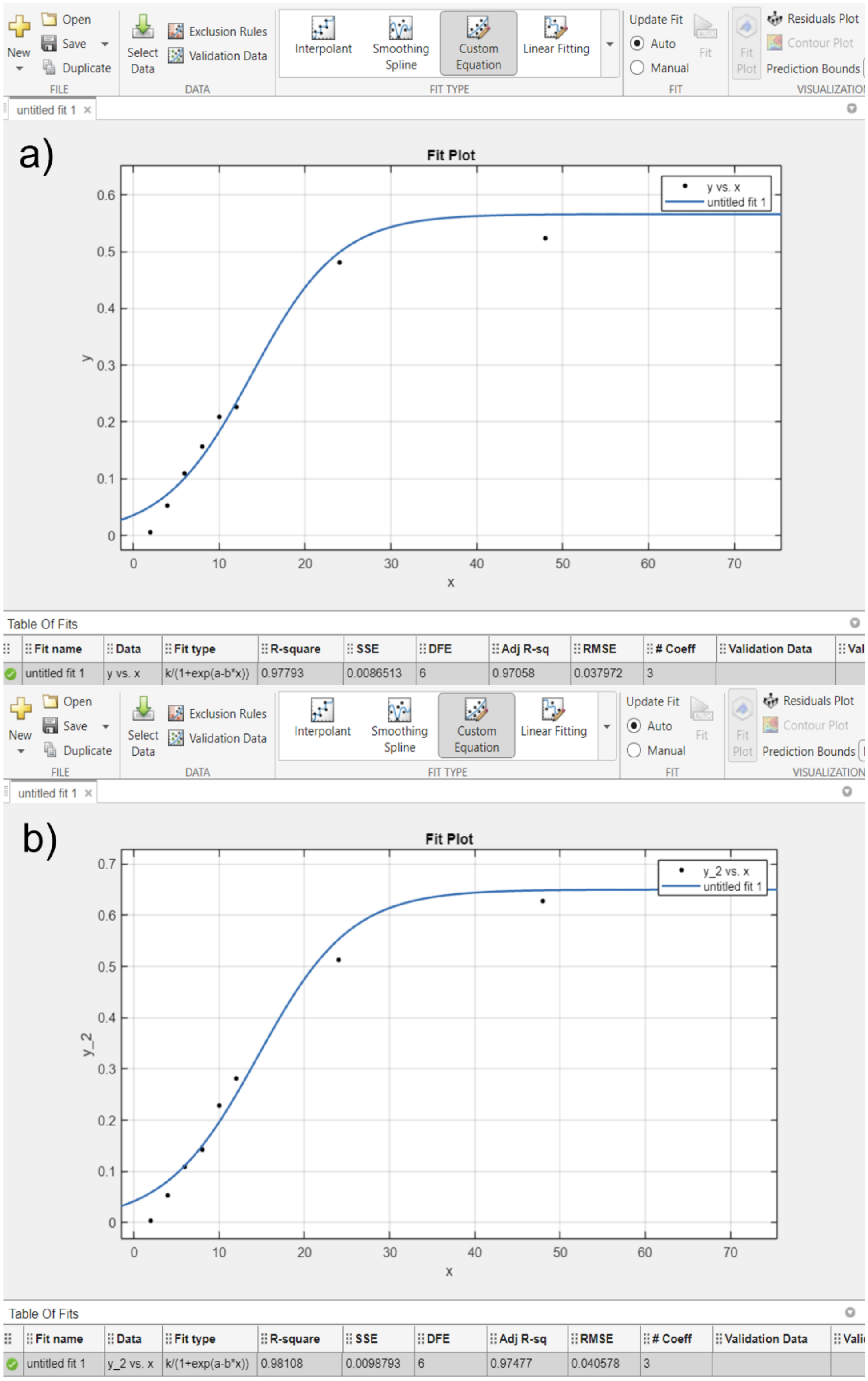

Fig. S6. The logistic fit of a) control, b) tested culture.

The absorbance scan of the medium containing pyocyanin standard was performed to verify if the measurements of the OD at 600 nm can also detect pyocyanin (Fig. S6.).

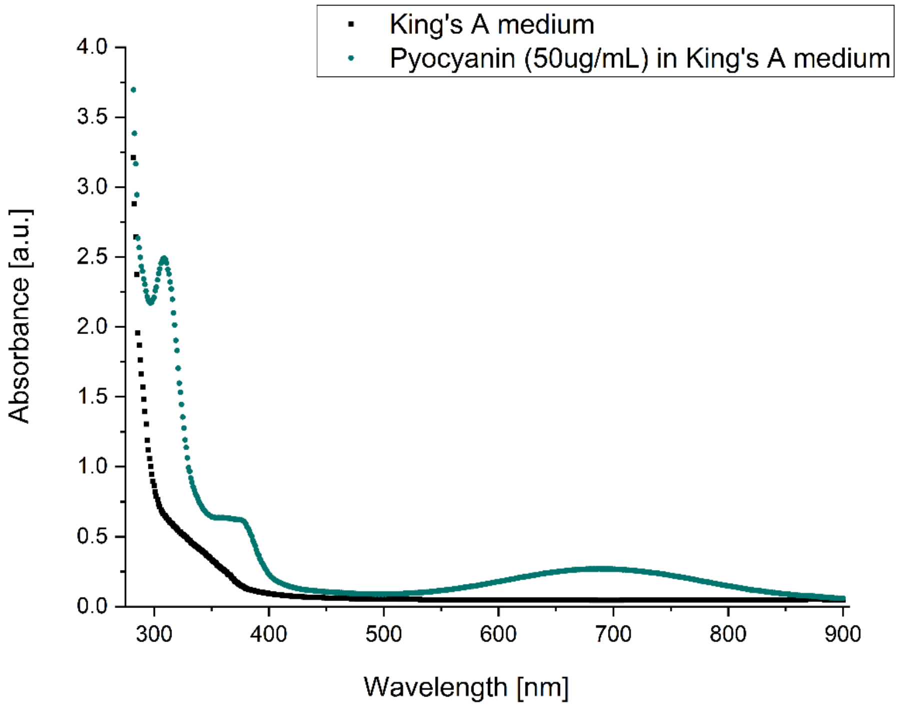

Fig. S7. Absorbance spectrum of pure King’s A medium and pyocyanin dissolved in King’s A medium.

The epifluorescence microscopy confirmed that the objects observed during the LIVE/DEAD assay were indeed bacteria. In Fig. S7. we present the exemplary images of LIVE/DEAD staining. The epifluorescence microscopy of the samples showed both viable and dead cells, with a big rise of the dead cells in control with gentamycin.

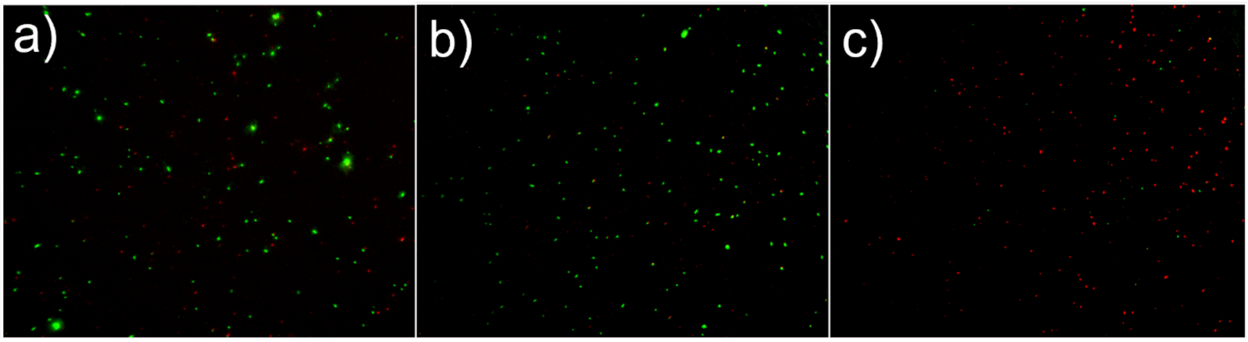

Fig. S8. Epifluorescence observations of the viability of a) the control, b) ZnO-supplemented culture, and c) gentamycin-treated culture.

In Fig. S9. we present the gating strategy for LIVE/DEAD staining.

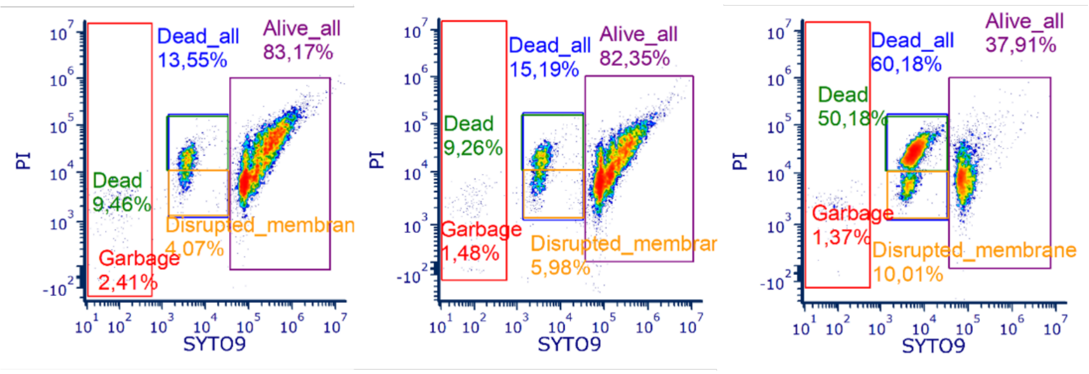

Fig. S9. Gating strategy for LIVE/DEAD assay (from left to right: the control, ZnO NP-treated sample, gentamycin sample).

Fig. S10. depicts the results of antibiotic susceptibility of the culture exposed to ZnO NPs and the control culture.

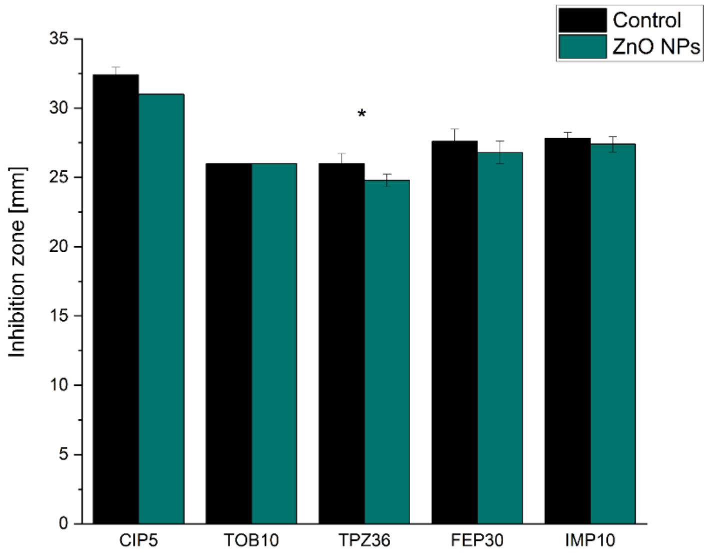

Fig. S10. Antibiotic susceptibility of *P. aeruginosa* control culture and culture incubated with 6.06 µg/mL of ZnO NPs for 6 hours.

S2.3. *Biofilm and eDNA studies*

The biofilm viability and biomass were quantified in the control, ZnO NPs-supplemented, and micrometric ZnO-supplemented culture. The results revealed few differences. Lower viability was noted in micrometric ZnO-supplemented culture at 24h and 72h of incubation (in comparison to the control). The biofilm biomass was significantly lower in the micrometric ZnO-supplemented culture after 48h and 72h, whereas the biomass of ZnO NPs supplemented culture was higher than in the control after 72h.

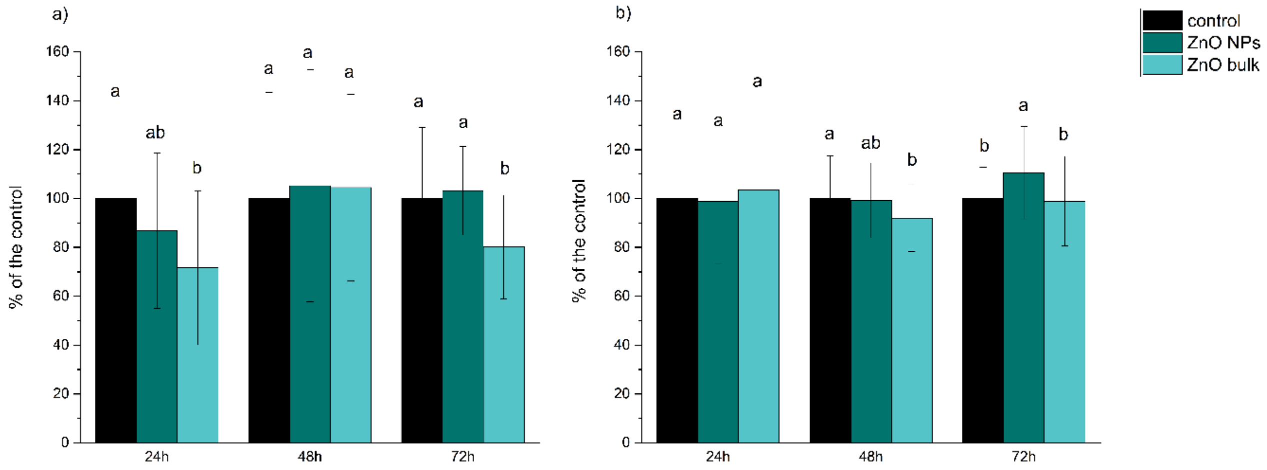

Fig. S11. Biofilm viability and biomass of the control culture, ZnO NP-supplemented culture, and micrometric ZnO.

The counts of viable cells in the control and ZnO NPs-supplemented culture showed a slightly higher number of viable cells in the case of the ZnO sample than in the control after 24 hours of incubation (Fig. S12.).

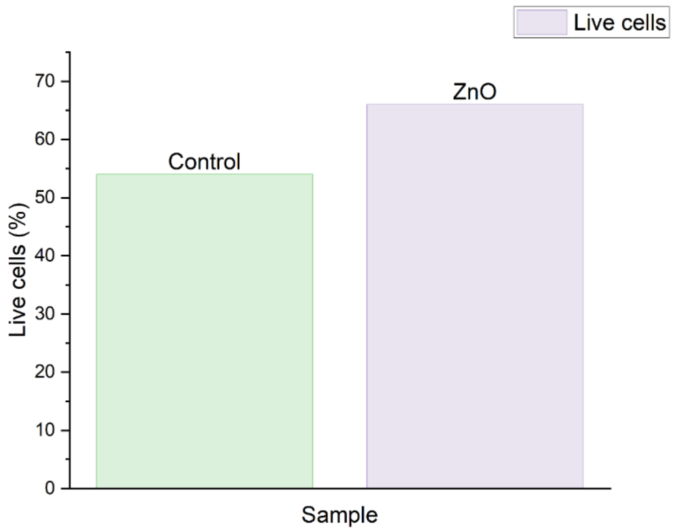

Fig. S12. The counts of viable cells in CLSM analysis.

The eDNA was quantified in the control, ZnO NPs-supplemented, and micrometric ZnO-supplemented culture. This analysis revealed that after 72 hours the content of eDNA is significantly higher in both ZnO NP-supplemented and micrometric ZnO-supplemented cultures. Moreover, such a trend was observed also after 24 and 48h. However, the differences were not statistically significant.

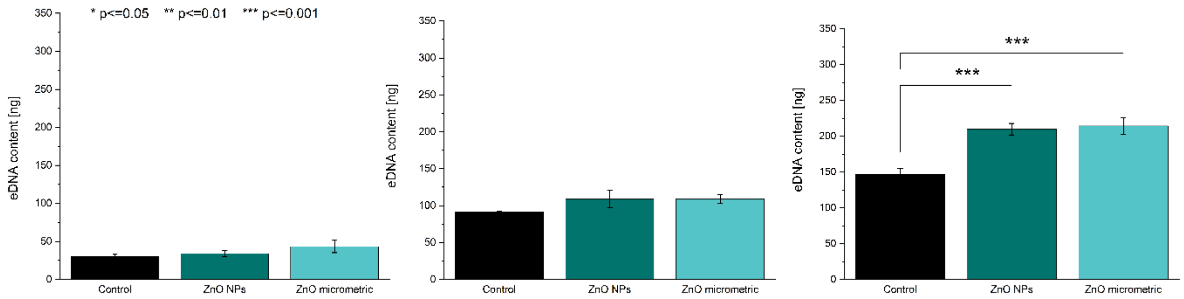

Fig. S13. eDNA concentration of the control culture, ZnO NP-supplemented culture and micrometric ZnO.

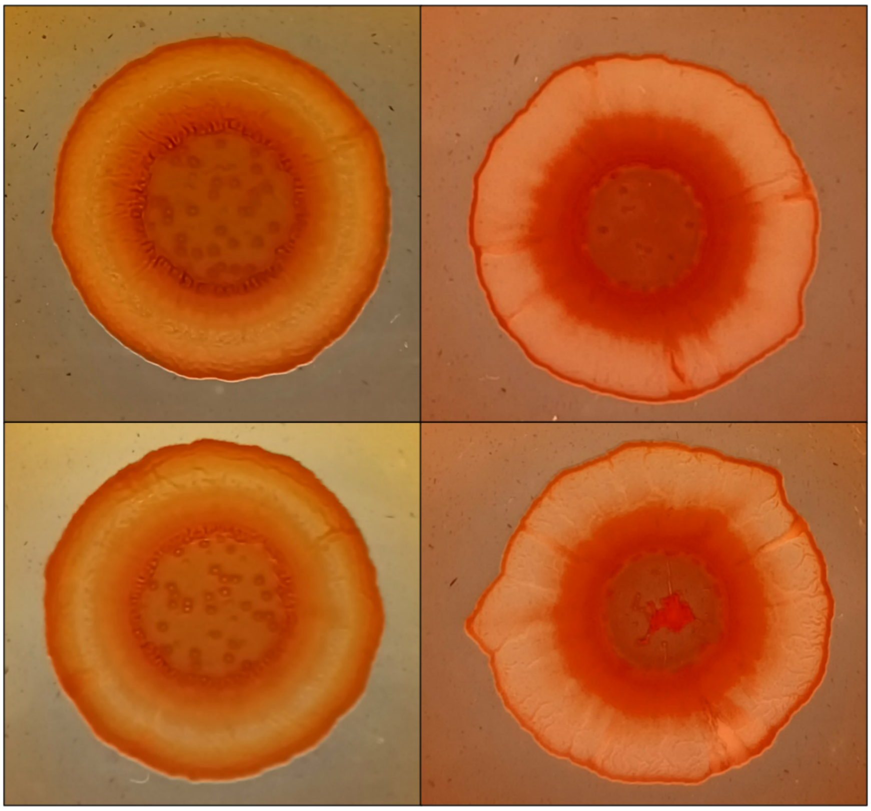

Fig. S14. Colony morphology a) control 25°C, b) control 32.6°C, c) ZnO-supplemented culture 25°C, d) ZnO-supplemented culture 32.6°C.

S2.4. *Stress analyses*

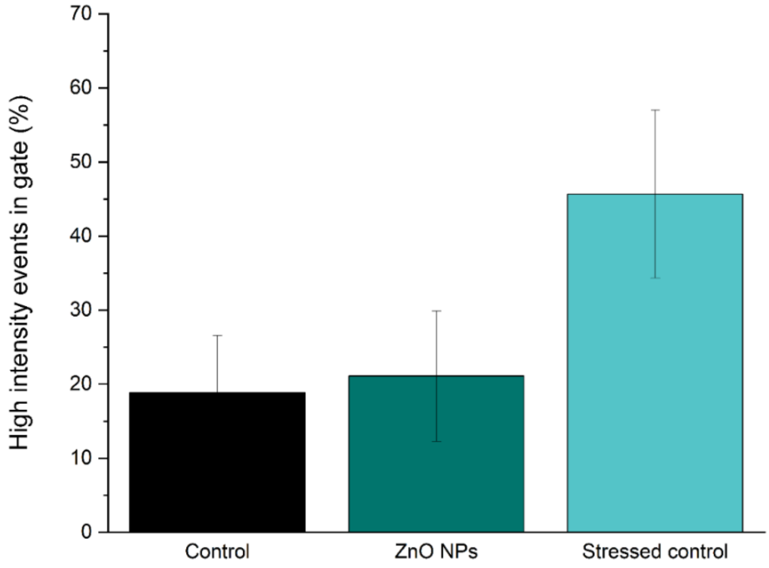

Fig. S15. CellROX Green analysis results after 6h of incubation.

S2.5. *Zinc content analysis*

ICP-OES analysis showed that the Zn content in the filtrates was at all times higher for the ZnO NPs samples than in micrometric ZnO samples (statistically significant differences were noted after 24 and 72h, Fig. S16.). This may suggest that more Zn^{2+} were released from the nanoparticles or that the nanoparticles passed the filter during the preparation of the samples.

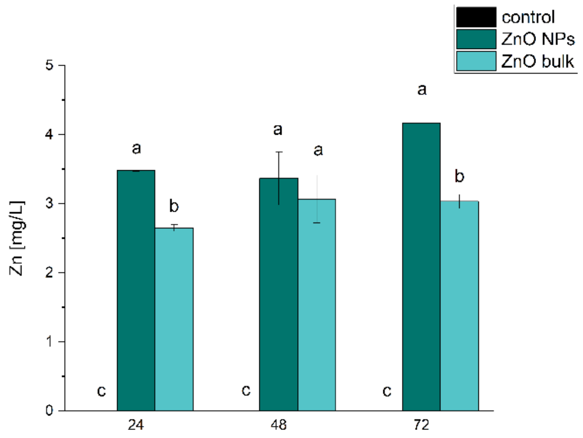

Fig. S16. ICP-OES analysis of the culture supernatants of zinc content in the control, ZnO NPs, and micrometric ZnO.
